# Effects of *Helleborus thibetanus* extract, a novel plant-derived molluscicide on *Oncomelania hupensis*, the intermediate host of *Schistosoma japonicum*

**DOI:** 10.1371/journal.pone.0354674

**Published:** 2026-09-11

**Authors:** Haonan Yu, Guoli Qu, Liping Wang, Jiankun Jin, Yuntian Xing

**Affiliations:** National Health Commission Key Laboratory of Parasitic Disease Control and Prevention, Jiangsu Provincial Key Laboratory on Parasite and Vector Control Technology, Jiangsu Institute of Parasitic Diseases, Wuxi, Jiangsu, China; Tongji University School of Medicine, CHINA

## Abstract

The snail, *Oncomelania hupensis*, is the only intermediate host of *Schistosoma japonicum*. Thus, snail control is one of the most rapid and effective strategies for disrupting schistosomiasis transmission. In this study, 125 botanical extracts were screened for molluscicidal activity, leading to the selection of the extract of *Helleborus thibetanus* (EHT) for further evaluation. EHT exhibited strong molluscicidal activity against *O. hupensis* (LC_50_ value of 12.54 mg/L and LD_50_ value of 1.400 g/m^2^). In field trials, an EHT dose of 10 g/m^2^ killed 85.19% of the snails. Histopathological evaluation revealed that EHT caused comprehensive damage to the cephalopodium, gill, salivary gland, stomach, and hepatopancreas. Importantly, EHT was also generally safe for non-target organisms, such as *Danio rerio*, *Daphnia magna*, *Pelophylax nigromaculatus*, *Cipangopaludina chinensis*, and *Neocaridina denticulata* at the test concentration. These results suggest the potential of EHT as an effective and eco-friendly molluscicide for the control of *O. hupensis*.

## 1. Introduction

Schistosomiasis remains one of the most pervasive parasitic diseases worldwide and ranks second to globally in terms of overall morbidity and socioeconomic burden [1]. The Global Burden of Disease Study estimates that schistosomiasis continues to affect nearly 70 countries and regions, placing more than 250 million people at risk [2,3]. *Oncomelania hupensis* is the only intermediate host of *Schistosoma japonicum* [4], making the control of *O. hupensis* populations a cornerstone strategy for reducing transmission and controlling endemicity [5].

Since the 1960s, niclosamide (NI) has been the World Health Organization’s (WHO) recommended molluscicide and remains the primary chemical agent used for snail control [6]. However, its broad-spectrum ecotoxicity, particularly toward non-target aquatic species, has raised substantial environmental concerns [7]. These issues are especially critical in China, where *O. hupensis* habitats overlap with key ecological regions of the Yangtze River Basin [5]. The implementation of the Yangtze River Protection Law in China, together with growing emphasis on ecological conservation, has made the search for safer molluscicidal alternatives increasingly urgent.

Botanical extracts have emerged as promising alternatives to synthetic molluscicides due to their generally favorable environmental profiles, rapid biodegradability, and lower potential for resistance development [8–11]. Plant-derived agents often contain multiple active compounds with diverse mechanisms of action, reducing the likelihood of resistance development. Furthermore, the vast diversity of natural flora provides an extensive resource for discovering new molluscicidal leads. As a result, identifying plant-based agents that couple strong activity with low ecological risk has become a focal point of current research.

*Helleborus thibetanus*, native to northwestern Sichuan and parts of Gansu and Shaanxi Provinces, is known for its antitumor and immunomodulatory activities and has traditional uses in treating urinary tract and inflammatory conditions [12]. Despite these documented properties, its potential molluscicidal activity has not been previously investigated.

Here, screening of 125 plant extracts identified the extract of *H. thibetanus* (EHT) as a potent molluscicidal candidate. Subsequent laboratory and field assessments demonstrated the efficacy of EHT against *O. hupensis*, along with its low toxicity to non-target organisms. These findings demonstrate the environmental compatibility of EHT, supporting its further development as a novel molluscicide targeting *O. hupensis*.

## 2. Materials and methods

### 2.1. Chemicals

All plant extracts were provided by Chengdu Lemeitian Pharmaceutical Technology Co., Ltd. All extracts were prepared following the manufacturer’s standardized procedure: dried plant materials were pulverized and extracted with water for 2 h, repeated three times. The combined filtrates were concentrated to yield a crude extract, which was then spray‑dried. Detailed quality control data are provided in the Supporting Information (S1 File). NI was sourced from MedChemExpress Co., Ltd. Dimethyl sulfoxide (DMSO), used to prepare extract stock solutions, was obtained from Zhejiang Baidi Biotechnology Co., Ltd.

### 2.2. Snail maintenance

*O. hupensis* snails were collected from Zhenjiang City, Jiangsu Province, China, and transported to the laboratory. The snails were rinsed three times with dechlorinated water and placed on plates for 24 h to allow free migration. Individuals that actively crawled out of the plates were considered highly active and transferred to large clean plates with filter paper at the bottom. The plates, each of which contained 1000 snails, were maintained at 25 ± 2 °C with a 12 h light/dark cycle. After adaptation for one week, active adult snails with 7–8 spirals were then randomly assigned to appropriate experimental groups for analysis of molluscicidal activity [11].

### 2.3. Assessment of molluscicidal efficacy

Preliminary screening was conducted using plate tests: A total of 50 g of soil was placed in 9-cm Petri dishes and moistened with 12–13 mL of dechlorinated water to create a mud plate. Ten snails were placed on each plate. The extracts (63 mg) were blended with flour using a ball mill and evenly distributed across the substrate via a fine-mesh sieve, with a final drug dosage of 10 g/m^2^ within the mud plate. The plates were covered with perforated lids to prevent the snails from escaping and were maintained at 26 °C for seven days. After treatment, the snails were collected, rinsed three times with dechlorinated water, and transferred to dry filter paper for 48 h of recovery. Survival was assessed using the knocking method. Snails that were only sprayed with flour represented the blank control, while the positive controls were treated with 2 g/m^2^ NI. The experiment was repeated three times.

Determination of the median lethal dose (LD_50_): Varying amounts of EHT (63, 31.5, 15.75, 7.88, and 3.94 mg) were blended with flour using a ball mill and then evenly spread over the mud plate using a fine-mesh sieve, resulting in final drug dosage of 10, 5, 2.5, 1.25, and 0.625 g/m^2^ on the plate. The plates were then treated in the same way as that used during the screening process. The samples were incubated in a chamber at 26 °C for 1, 3, and 7 days, after which mortality was assessed as described above. Plates that had been sprayed with flour only served as the blank controls, while those treated with NI (1, 0.5, 0.25, 0.125, and 0.0625 g/m^2^) represented the positive controls. The experiment was repeated three times. LD_50_ values of EHT and NI were calculated separately based on the experimental results.

Assessment of the median lethal concentration (LC_50_): EHT was dissolved in DMSO to 40 mg/mL to prepare a stock solution. This solution was then diluted with dechlorinated water to working solutions of 80, 40, 20, 10, 5, 2.5, and 1.25 mg/L. One-hundred-milliliter aliquots of these solutions were placed in beakers, and 10 snails were immersed in each beaker for 24, 48, and 72 h, after which mortality was assessed as above. Dechlorinated water containing 0.01% (*v/v*) DMSO served as the blank control and dilutions of NI (1, 0.5, 0.25, 0.125, 0.0625, and 0.03125 mg/L) represented positive controls. The experiment was repeated three times. LC_50_ values of EHT and NI were calculated separately based on the experimental results [13].

### 2.4. Histopathology

Histological analyses were carried out after snails were exposed for 72 h to EHT at 1/2 LC_50_ concentration. Tissues were dissected immediately after treatment and fixed in 4% paraformaldehyde, followed by dehydration through a graded ethanol series. Samples were then cleared in xylene and embedded in paraffin. Serial sections were cut at 5 µm thickness using a rotary microtome, with three replicate sections prepared for each tissue type. Slides were stained with hematoxylin and eosin (H&E) to allow detailed microscopic evaluation. Morphological alterations were examined using a light microscope following established histopathological criteria [14].

### 2.5. Evaluation of toxicity to non-target organisms

The EHT stock solutions were diluted with dechlorinated water to final test concentrations of 10, 5, 2.5, 1.25, and 0.625 mg/L. NI served as the positive control, with 5–7 concentrations selected on the basis of preliminary range-finding tests. Negative controls consisted of dechlorinated water containing 0.01% (*v/v*) DMSO. Mortality was recorded every 24 h for 72 h, and deceased individuals were removed promptly. All treatments were performed in triplicate. The LC_50_ values for each non-target species after EHT treatment were calculated from the results.

#### 2.5.1. Assessment of acute toxicity in *Danio rerio.*

*D. rerio* were obtained from a commercial vendor and acclimated for one month in dechlorinated water (pH 7.2 ± 0.2; dissolved oxygen 6.0 ± 0.4 mg/L; hardness 68–78 mg/L as CaCO_3_) at 25 ± 2 °C under a 16:8 h light: dark cycle. Fish were fed a commercial diet daily. Acute toxicity assays were conducted in 500-mL glass beakers, each containing ten fish. Mortality was defined as the lack of movement within 5 s following gentle stimulation with forceps [15,16].

#### 2.5.2. Assessment of acute toxicity in *Daphnia magna.*

*D. magna* were purchased from a commercial source and maintained in dechlorinated water (pH 7.2–7.4; oxygen saturation 88–97%; hardness 68–78 mg/L as CaCO_3_) at 25 ± 2 °C with a 16:8 h light: dark cycle. Cultures were refreshed weekly and fed twice weekly with *Chlorella* spp. and yeast. After two months of culture, neonates from a single parthenogenetic female were selected for testing. Groups of 10 neonates were placed in 100-mL beakers. Mortality was recorded as the absence of spontaneous movement within 15 s of gently agitating the vessel [17,18].

#### 2.5.3. Assessment of acute toxicity in *Pelophylax nigromaculatus.*

Tadpoles of *P. nigromaculatus* were acquired commercially and acclimated for two weeks in dechlorinated water (pH 6.0–8.5; oxygen saturation > 60%; hardness 10–250 mg/L as CaCO_3_) at 25 ± 2 °C under a 16:8 h light: dark cycle. Water was replaced twice weekly and animals were fed commercial tadpole feed. Acute toxicity trials were performed in 500-mL beakers, with ten tadpoles per beaker. Mortality was defined as the absence of tail or body movement within 5 s following gentle tactile stimulation [19].

#### 2.5.4. Assessment of acute toxicity in *Cipangopaludina chinensis.*

*C. chinensis* specimens were collected from Taihu Lake (Wuxi, Jiangsu Province), cleaned thoroughly, and acclimated for one month in dechlorinated water (pH 7.2 ± 0.2; dissolved oxygen 6.0 ± 0.4 mg/L; hardness 68–78 mg/L as CaCO_3_) at 25 ± 2 °C with a 16:8 h light: dark cycle. Water was renewed biweekly, and snails were fed *Chlorella* spp. Individuals with shell heights of 15 ± 2 mm were selected for testing in 500-mL beakers (ten snails per beaker). Mortality was recorded when no muscular contraction occurred following stimulation of exposed soft tissue with a steel needle [20].

#### 2.5.5. Assessment of acute toxicity in *Neocaridina denticulata.*

*N. denticulata* were purchased from a commercial provider and acclimated for one month in dechlorinated water (pH 7.4–7.8; dissolved oxygen > 7.3 mg/L; hardness 38–45 mg/L as CaCO_3_) at 25 ± 2 °C under a 16:8 h light: dark cycle. Aquatic plants were included as both forage and shelter, and water was replaced weekly. Active shrimps were selected for testing in 500-mL beakers (n = 10 per beaker). Mortality was defined as the absence of response to tactile stimulation with forceps [21,22].

### 2.6. Field molluscicidal trials

**Fig 1:**
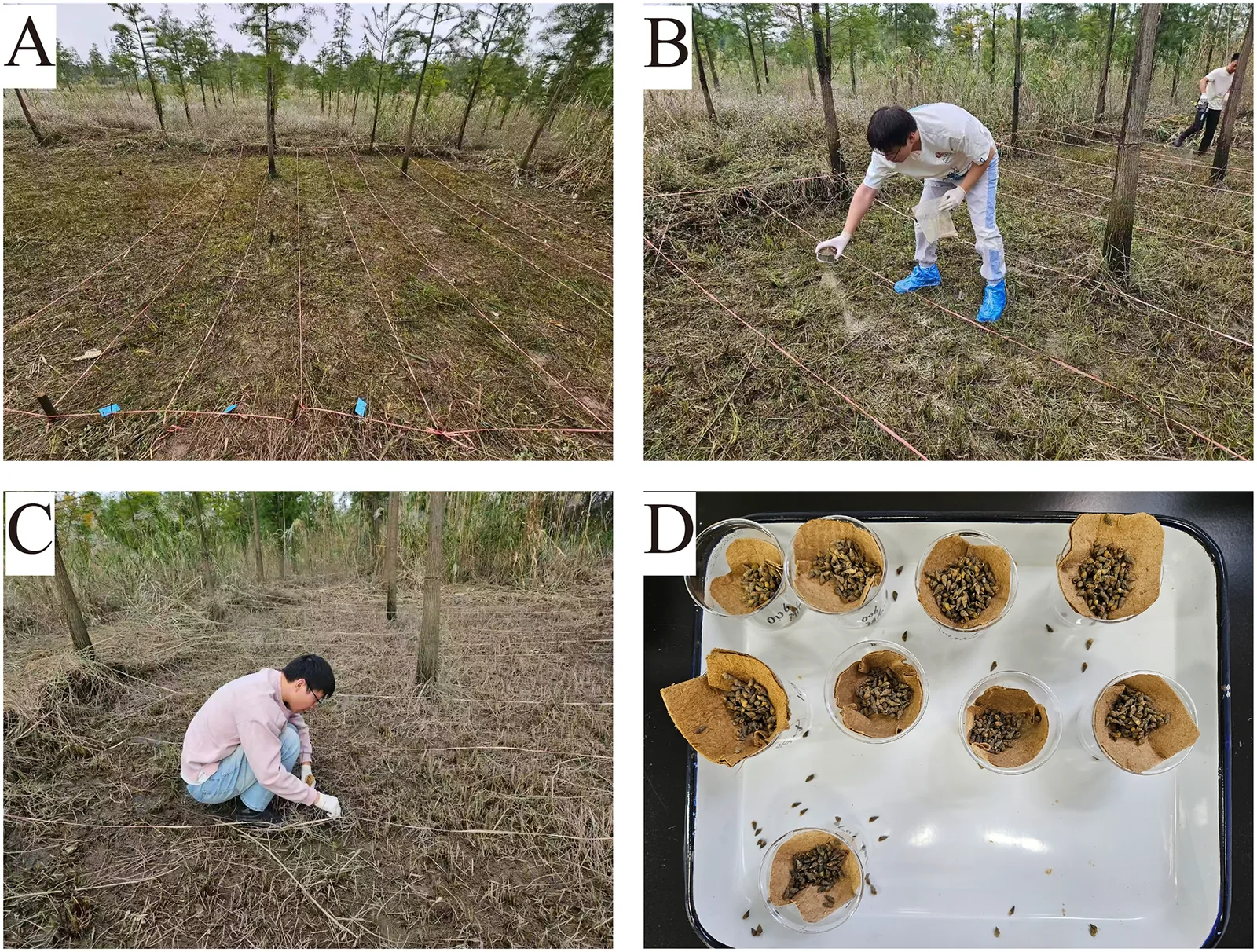
Field molluscicidal trials. **Note:** A: Area allocation; B: Drug application; C: Snail sampling; D: 48-h recovery of the sampled snails.

Field assessments were conducted along the Yangtze River (32.20° N, 119.58° E) in Zhenjiang City, Jiangsu Province, China, where *O. hupensis* were abundant (> 10 snails per frame, 33 cm × 33 cm). The experimental site consisted of moist soil with sparse vegetation. Experimental plots measuring 1 m × 10 m were delineated prior to treatment (Fig 1). The individual pictured in Fig 1 has provided written informed consent (as outlined in PLOS consent form) to publish their image. EHT was applied at 10, 7.5, and 5 g/m^2^. NI (2 g/m^2^) served as the positive control, and an untreated plot served as the negative control. At 1, 3, and 7 days after application, 100 snails were obtained at random from each plot, transported to the laboratory, rinsed with dechlorinated water, and maintained on dry filter paper for 48 h before survival evaluation [13].

### 2.7. Data analysis

Data related to molluscicidal activity and non-target toxicity were compiled and processed using Microsoft Excel 2020. Statistical analyses were performed with GraphPad Prism 8.0.2 and SPSS 27.0. Mortality data on the molluscicidal activity of plant extracts against *O. hupensis* are expressed as mean ± standard deviation (SD). The molluscicidal activity of EHT against *O. hupensis* (LC_50_ and LD_50_) was determined using probit analysis [log(inhibitor) vs. normalized response -- variable slope] and expressed as LC_50_ or LD_50_ values with 95% confidence intervals (95% CI). The molluscicidal activities of 125 plant extracts were analyzed using *t*-tests, comparing EHT activity with the activities of other extracts. The results of the field trials results were analyzed by Bonferroni-corrected χ^2^ tests. *P* < 0.05 was considered statistically significant.

## 3. Results

### 3.1. EHT exhibits potent molluscicidal activity

**Fig 2:**
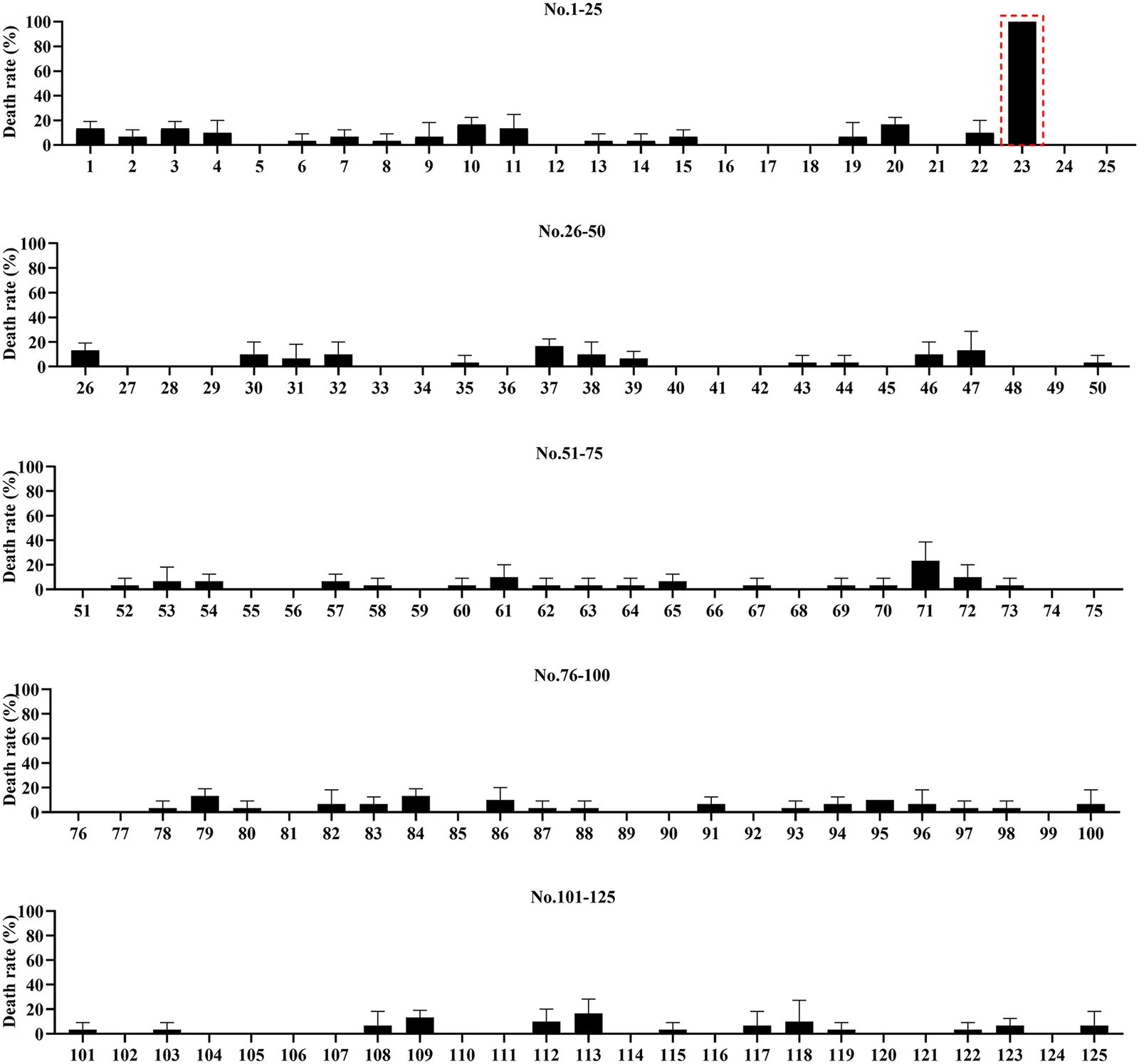
Molluscicidal activity of 125 plant extracts against *O. hupensis.* **Note:** The results represent the average of three replicates ± SD. No.23 (extract of *H. thibetanus*, EHT) shows the highest molluscicidal activity of the 125 plant extracts (*t*-test, *P* < 0.0001, compared with any plant extract). The positive control was 2 g/m^2^ niclosamide, while the blank control was flour. The mortality rates of *O. hupensis* in the positive and blank controls were 100.00% and 0.00%, respectively.

The initial screening of the molluscicidal activities of 125 plant extracts against *O. hupensis* indicated that EHT exhibited the highest potency (S1 Table). At the test dosage of 10 g/m^2^, EHT treatment resulted in 100.00 ± 0.00% snail mortality within seven days, whereas the other extracts showed markedly less activity, with mortality rates below 30% (Fig 2).

EHT yielded a LC_50_ value of 12.54 mg/L at 72 h, as shown the immersion test, and a LD_50_ value of 1.400 g/m^2^ at 7 d, as indicated by the plate test (Table 1).

**Table 1:** Molluscicidal efficacy of EHT against *O. hupensis.*

Drug	Method	Treatment time (d)	Toxicity equation	R squared	LC_50_/LD_50_ (mg/L or g/m^2^, 95% CI)
EHT	Immersion	1	Y = 100/(1 + 10^(1.843-x) *1.516^)	0.9247	69.68 (51.72 to 123.9)
		2	Y = 100/(1 + 10^(1.433-x) *1.477^)	0.9728	27.12 (21.12 to 35.39)
		3	Y = 100/(1 + 10^(1.098-x) *2.691^)	0.9906	12.54 (10.79 to 14.67)
	Plate	1	—	—	> 10.00
		3	Y = 100/(1 + 10^(0.4659-x) *0.8259^)	0.9884	2.923 (2.381 to 3.613)
		7	Y = 100/(1 + 10^(0.1462-x) *1.827^)	0.9689	1.400 (0.9567 to 1.975)
NI	Immersion	1	Y = 100/(1 + 10^(−1.026-x) *7.038^)	0.9995	0.09425 (0.09284 to 0.09565)
		2	Y = 100/(1 + 10^(−1.054-x) *5.925^)	0.9993	0.08840 (0.08668 to 0.09011)
		3	Y = 100/(1 + 10^(−1.119-x) *4.392^)	0.9811	0.07608 (0.06681 to 0.08556)
	Plate	1	Y = 100/(1 + 10^(−0.4854-x) *2.801^)	0.9975	0.3271 (0.2974 to 0.3601)
		3	Y = 100/(1 + 10^(−0.5074-x) *3.057^)	0.9833	0.3109 (0.2414 to 0.3931)
		7	Y = 100/(1 + 10^(−0.5191-x) *3.106^)	0.9721	0.3026 (0.2130 to 0.4083)

**Note:** EHT: extract of *H. thibetanus*. Niclosamide (NI) represented the positive control. Dechlorinated water with 0.01% (*v/v*) DMSO and flour represented the blank controls in the immersion and plate tests, respectively. Snail mortalities were 0% in the blank controls in both the plate test (1, 3, and 7 days) and immersion test (1, 2, and 3 days).

### 3.2. EHT damages to multiple *O. hupensis* organs

**Fig 3:**
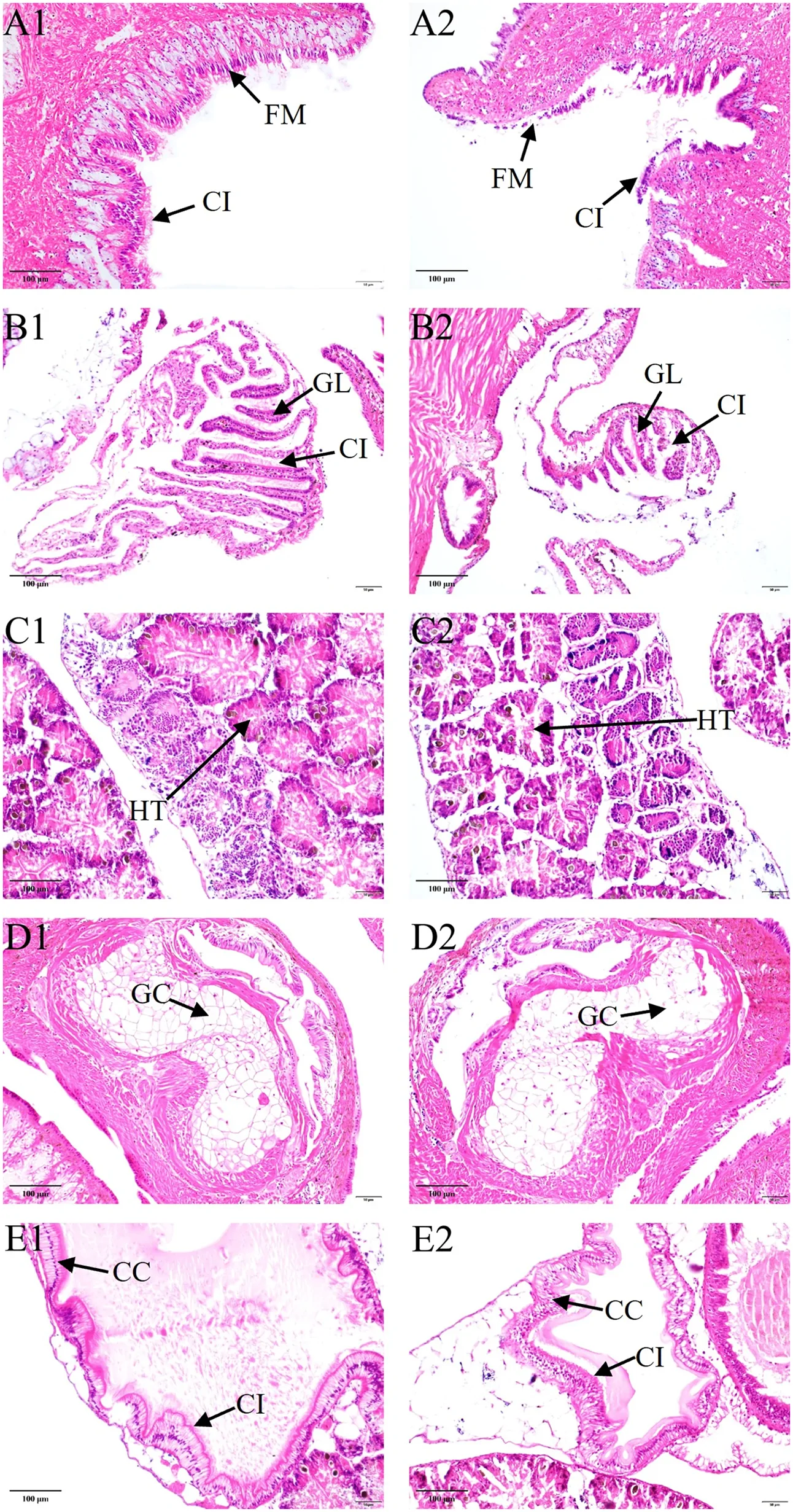
Histopathological changes in *O. hupensis* following EHT exposure. **Note:** EHT: extract of *H. thibetanus*. A1, B1, C1, D1 and E1 represent control snails; A2, B2, C2, D2 and E2 represent EHT-treated snails. (A) Head-foot; (B) Gill; (C) Hepatopancreas; (D) Salivary gland; (E) Stomach. FM: foot-sole membrane; CI: cilia; GL: gill leaflet; HT: hepatic tubule; GC: gland cell; CC: columnar epithelial cell.

As shown in Fig 3, EHT treatment resulted in marked damage to multiple organs of *O. hupensis*. Snails in the control group exhibited normal tissue architecture: the foot sole membrane maintained sharply defined margins and dense ciliation (Fig 3, A1); gill leaflets possessed intact surface cilia securely attached to the gill sac (Fig 3, B1); hepatic tubules in the hepatopancreas displayed characteristic stellate lumina (Fig 3, C1); salivary gland cells appeared vesiculated with irregular circular profiles (Fig 3, D1); and gastric epithelium consisted of orderly columnar cells with distinct cell borders and abundant apical cilia (Fig 3, E1). After exposure to EHT, the foot sole membrane became disrupted with near-complete loss of cilia (Fig 3, A2); gill leaflets were fragmented and detached from the gill sac (Fig 3, B2); hepatic tubule lumina collapsed into amorphous debris (Fig 3, C2); salivary gland cells underwent extensive cytolysis (Fig 3, D2); and gastric columnar cells lost structural organization, showing poorly defined boundaries and substantial ciliary shedding (Fig 3, E2).

### 3.3. EHT shows low toxicity to non-target organisms

The toxicity of EHT to non-target organisms was dose- and time-dependent (Table 2). After 72 h of treatment, the LC_50_ values were 7.481, 3.671, 1.346, > 10, and > 10 mg/L for *D. rerio*, *D. magna*, *P. nigromaculatus*, *C. chinensis* and *N. denticulata*, respectively. In contrast, NI showed substantially higher toxicity, with LC_50_ values of 0.1097, 0.2745, 0.03427, 0.07725, and > 10 mg/L, respectively, at 72 h.

**Table 2:** EHT toxicity to non-target organisms.

Species	Treatment time (h)	Drug	Toxicity equation	R squared	LC_50_ (mg/L, 95% CI)
*Danio rerio*	24	EHT	—	—	> 10
		NI	Y = 100/(1 + 10^(−0.8411-x) *4.929^)	0.9997	0.1442 (0.1397 to 0.1489)
	48	EHT	Y = 100/(1 + 10^(0.9468-x) *3.338^)	0.9992	8.846 (8.573 to 9.129)
		NI	Y = 100/(1 + 10^(−0.9323-x) *3.631^)	0.9904	0.1169 (0.09912 to 0.1351)
	72	EHT	Y = 100/(1 + 10^(0.874-x) *3.524^)	0.9989	7.481 (7.153 to 7.822)
		NI	Y = 100/(1 + 10^(−0.9598-x) *3.671^)	0.9951	0.1097 (0.09802 to 0.1216)
*Daphnia magna*	24	EHT	—	—	> 10
		NI	Y = 100/(1 + 10^(−0.4517-x) *3.573^)	0.9972	0.3535 (0.3203 to 0.3900)
	48	EHT	Y = 100/(1 + 10^(0.8685-x) *2.449^)	0.9945	7.388 (6.643 to 8.267)
		NI	Y = 100/(1 + 10^(−0.5076-x) *4.259^)	0.9785	0.3108 (0.2527 to 0.4005)
	72	EHT	Y = 100/(1 + 10^(0.5648-x) *2.186^)	0.9909	3.671 (3.047 to 4.449)
		NI	Y = 100/(1 + 10^(−0.5614-x) *3.171^)	0.9750	0.2745 (0.2009 to 0.3640)
*Pelophylax nigromaculatus*	24	EHT	—	—	> 10
		NI	Y = 100/(1 + 10^(−1.243-x) *4.703^)	0.9990	0.05709 (0.05498 to 0.05912)
	48	EHT	Y = 100/(1 + 10^(0.1289-x) *1.551^)	0.9227	1.346 (0.6656 to 2.363)
		NI	Y = 100/(1 + 10^(−1.377-x) *4.710^)	0.9999	0.04196 (0.04159 to 0.04233)
	72	EHT	Y = 100/(1 + 10^(0.008727-x) *2.730^)	0.8832	1.020 (0.6545 to 2.001)
		NI	Y = 100/(1 + 10^(−1.465-x) *4.401^)	1.000	0.03427 (0.03412 to 0.03442)
*Cipangopaludina chinensis*	24	EHT	—	—	> 10
		NI	Y = 100/ (1 + 10^(−0.2107-x) *1.149^)	0.8499	0.6156 (0.2908 to 1.460)
	48	EHT	—	—	> 10
		NI	Y = 100/(1 + 10^(−0.8253-x) *1.685^)	0.9896	0.1495 (0.1249 to 0.1792)
	72	EHT	—	—	> 10
		NI	Y = 100/(1 + 10^(−1.112-x) *2.21^)	0.9657	0.07725 (0.05946 to 0.1025)
*Neocaridina denticulata*	24	EHT	—	—	> 10
		NI	—	—	> 10
	48	EHT	—	—	> 10
		NI	—	—	> 10
	72	EHT	—	—	> 10
		NI	—	—	> 10

**Note:** EHT: extract of *H. thibetanus*. Niclosamide (NI) represents the positive control. Dechlorinated water with 0.01% (*v/v*) DMSO represents the blank control. The mortality rates of non-target organisms were 0% in the blank control at 24, 48, and 72 h.

### 3.4. EHT is effective for snail control in field trials

**Fig 4:**
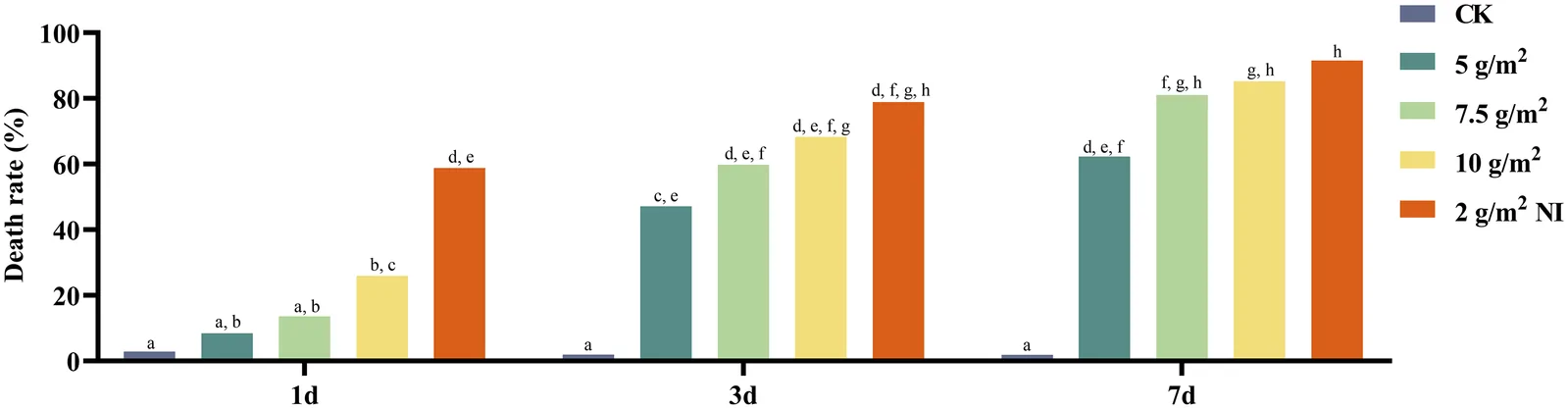
Efficacy of EHT against *O. hupensis* in the field. **Note:** EHT: extract of *H. thibetanus*. Niclosamide (2 g/m^2^) was used as the positive control and flour as the blank control. Different letters in columns indicate significant differences (Bonferroni-corrected χ^2^ test, *P* < 0.05).

Field trials demonstrated dose- and time-dependent molluscicidal efficacy of EHT, consistent with the laboratory results (Fig 4). At application doses of 5, 7.5, and 10 g/m^2^, the mortality rates were 8.49, 13.64, and 25.96%, respectively, on day 1, 47.12, 59.80, and 68.27%, respectively, on day 3, and 62.26, 81.00, and 85.19%, respectively on day 7. The mortality rates of *O. hupensis* in the positive and blank controls after 1, 3, and 7 days were 58.82, 78.90, and 91.51% and 2.86, 1.92, and 1.90%, respectively.

## 4. Discussion

Although niclosamide exhibits high molluscicidal activity, it is also highly toxic to non-target organisms. Increasing public environmental awareness and the enactment of the Yangtze River Protection Law in China have led to marked reductions in its application, thereby creating an urgent need for novel, environmentally friendly molluscicides. In recent years, interest in plant-based molluscicides has accelerated due to their favorable environmental profiles, positioning botanical compounds as strong contenders for future snail-control strategies [23–26]. Numerous studies have evaluated the efficacy of plant extracts or active ingredients against *O. hupensis* [27]. However, few studies have assessed molluscicidal activity using plate tests. In this study, EHT was found to be the most effective of 125 plant extracts using plate tests (Fig 2) and was subsequently evaluated as a potential plant-derived molluscicide against *O. hupensis*. Spray bioassays yielded 3- and 7-day LD_50_ values of 2.923 g/m^2^ and 1.400 g/m^2^, respectively (Table 1), both satisfying the registration threshold (LD_50_ < 10 g/m^2^) as outlined in the Efficacy Test Methods and Evaluation of Molluscicides for Pesticide Registration (NY/T 1617–2008) [28]. In contrast to aquatic intermediate-host snails (e.g., *Biomphalaria spp.* and *Bulinus spp.*), *O. hupensis* has an amphibious life history where adults are predominantly terrestrial, living in environments such as moist soil and vegetation, while juveniles develop in water. The relatively modest performance in immersion assays (72 h LC_50_ = 12.54 mg/L, Table 1) does not compromise the findings from the field experiments as > 90% of targeted snails during control campaigns are terrestrial adults, in contrast to fully aquatic species where immersion efficacy is critical.

The gills are the major organs used by *O. hupensis* for respiration, and are composed of gill filaments with a central axis attached to the filaments [29]. The gill filaments contain numerous cilia, which provide mechanical support for the gill, enabling full expansion of the gill lobes for optimal oxygen capture, as well as drive water through the gaps between the filaments in opposite directions to the blood circulation for greater oxygen delivery [30]. The hepatopancreas is central to both detoxification and nutrient metabolism in mollusks [31]. In this study, we found that EHT treatment caused widespread damage to *O. hupensis*, including injuries to the gills, hepatopancreas, salivary glands, stomach, and foot (Fig 3), many of which are organs integral to digestion and locomotion, thereby further compromising survival. These effects are similar to those reported in *O. hupensis* following exposure to linalool [32]. Collectively, the observed tissue breakdown likely underlies the mortality associated with EHT exposure.

In addition to molluscicidal activity, toxicity to non-target organisms represents a critical criterion for pesticide registration. Accordingly, this study further evaluated the toxicity of EHT to non-target organisms. The five non-target organisms assessed in this study included *D. rerio* [33,34] (fish), *D. magna* [35,36] (zooplankton), *P. nigromaculatus* [37] (amphibians), *C. chinensis* [38,39] (benthic fauna), and *N. denticulata* [40,41] (crustaceans), all of which are ecologically important sentinel species in aquatic toxicology due to their sensitivity to pollutants. EHT exhibited comparatively low toxicity in all tested species (Table 2). Its 72-h LC_50_ values for *D. rerio* and *D. magna* were 7.481 mg/L and 3.671 mg/L, accounting for only 1.40% and 7.48% of the toxicity of NI, respectively. EHT was also far less toxic to *D. rerio* than the commercial botanical molluscicide “Luowei” (72-h LC_50_ = 0.15 mg/L), representing merely 2.01% of its toxicity [42]. This markedly lower hazard is an important strength in light of the Yangtze River Protection Law in China and increasingly stringent environmental regulations, which contrasts with the broad-spectrum toxicity of NI, including its propensity to cause large-scale fish and amphibian mortality [43,44].

Field evaluation is critical for determining whether a candidate agent can transition into a commercial molluscicide. In field trials on Yangtze River beach, EHT at 7.5 g/m^2^ and 10 g/m^2^ produced > 80% *O. hupensis* mortality within 7 days (Fig 4). According to the NY/T 1617–2008 guideline, these results satisfy the required threshold for registration and thus support advancement of EHT for further development in China [28].

Despite these promising findings, several limitations warrant consideration. For example, subsequent efforts should prioritize the isolation and structural elucidation of the major bioactive constituents of EHT, which can lead to further enhancement of its molluscicidal potency. Thereafter, expanded field trials across diverse schistosomiasis-endemic settings would be indispensable for verifying the robustness of the observed efficacy under heterogeneous environmental conditions. Furthermore, comprehensive, long-term ecotoxicological assessments, including sub-lethal endpoints, chronic exposure scenarios, and resistance monitoring, will be required to ensure both regulatory compliance and the sustainable deployment of this intervention [45].

## 5. Conclusion

EHT exhibited potent molluscicidal activity while maintaining a favorable environmental safety profile. Histopathological examination revealed extensive damage to multiple organs of *O. hupensis*. The field test confirmed that EHT meets the molluscicide registration criteria, underscoring its practical applicability. Collectively, EHT showed strong molluscicidal ability and minimal toxicity, indicating its potential for the development of novel molluscicides against *O. hupensis*.

## Supporting information

S1 Table Molluscicidal activity of 125 plant extracts. (DOCX)

S1 File Certificate of Analysis (COA) report of 125 plant extracts. (PDF)

S2 File Raw data. (XLSX)
