# Supplementary material for: Effects of Helleborus thibetanus extract, a novel plant-derived molluscicide on Oncomelania hupensis, the intermediate host of Schistosoma japonicum
Source: PLoS One. 2026 Sep 11;21(9):e0354674. doi: 10.1371/journal.pone.0354674 (PMC13567751; doi:10.1371/journal.pone.0354674)
Supplement: Supplementary file 1 [file pone.0354674.s002.pdf]

Effects of *Helleborus thibetanus* extract, a novel plant-derived molluscicide on *Oncomelania hupensis*, the intermediate host of *Schistosoma japonicum*

Haonan Yu

Effects of *Helleborus thibetanus* extract, a novel plant-derived molluscicide on *Oncomelania hupensis*, the intermediate host of *Schistosoma japonicum*

Haonan Yu [1], Guoli Qu [1], Liping Wang [1], Jiankun Jin [1], Yuntian Xing[1,*]

[1] National Health Commission Key Laboratory of Parasitic Disease Control and Prevention, Jiangsu Provincial Key Laboratory on Parasite and Vector Control Technology, Jiangsu Institute of Parasitic Diseases, Wuxi, Jiangsu, China

[*] Corresponding author

E-mail: xingyuntian@jipd.com (YTX).

This file includes the Certificate of Analysis (COA) report of 125 plant extracts

[1], Guoli Qu [1], Liping Wang [1], Jiankun Jin [1], Yuntian Xing[1,*]

[1] National Health Commission Key Laboratory of Parasitic Disease Control and Prevention, Jiangsu Provincial Key Laboratory on Parasite and Vector Control Technology, Jiangsu Institute of Parasitic Diseases, Wuxi, Jiangsu, China

[*] Corresponding author

E-mail: xingyuntian@jipd.com (YTX).

This file includes the Certificate of Analysis (COA) report of 125 plant extracts

*No.*1 *Rhodiola rosea* extract

**Product Name**	*Rhodiola rosea* extract	
Lot Number	T0089	
Quantity	500 kg	
Manufacture Date	2015-12-03	
Testing Date	2015-12-04	
**Analysis**	**Specification**	**Results**
Appearance	Brown-red fine powder	Complies
Pesticides	Negative	Complies
Residual solvent	Eur. Pharm.	Complies
Ash	≤ 5.0%	2.5%
Moisture	≤ 5.0%	3.12%
Heavy metals	≤ 10 ppm	Complies
Pb	≤ 2.0 ppm	Complies
As	≤ 2.0 ppm	Complies
Hg	≤ 0.5 ppm	Complies
Odor	Characteristic	Complies
Particle size	100% through 80 mesh	Complies
**Microbiological**		
Total of bacteria	≤1000 cfu/g	Complies
Fungi	≤100 cfu/g	Complies
Salmonella	Negative	Complies
E. coli	Negative	Complies
Storage	Store in cool & dry place. Do not freeze.<br>Keep away from strong light and heat.	
Shelf life	2 years when properly stored.	

No.2 Pausinystalia yohimbe extract

**Product Name**	*Pausinystalia yohimbe* extract		
Lot Number	T0236		
Quantity	580 kg		
Manufacture Date	2022-05-16		
Testing Date	2022-05-18		
**Analysis**	**Specification**	**Results**	
Appearance	Brown-yellow powder	Complies	TLC
Odor	Characteristic	Characteristic	TLC
Taste	Characteristic	Characteristic	TLC
Extract Excipient	None	None	TLC
Particle size	100% through 80 mesh	Complies	TLC
Sulphated Ash	≤ 5.0%	3.71%	TLC
Loss on Drying	≤ 5.0%	3.63%	TLC
Bulk Density	40 - 60 g/100 mL	49.5 g/100 mL	TLC
**Heavy Metals**			
Heavy metals	10 ppm Max	Complies	Atomic Absorption
Pb	1.0 ppm Max	0.1 ppm Max	Atomic Absorption
As	1.0 ppm Max	0.2 ppm Max	Atomic Absorption
Hg	0.1 ppm Max	0.02 ppm Max	Atomic Absorption
Cd	1.0 ppm Max	0.1 ppm Max	Atomic Absorption
Pesticide Residues	1.0 ppm Max	Complies	Gas Chromatography
**Microbiological**			
Total plate count	1000 cfu/g Max	Complies	AOAC
Yeast & Mold	100 cfu/g Max	Complies	AOAC
E. coli	Negative	Negative	AOAC
Salmonella	Negative	Negative	AOAC
Staphylococcus	Negative	Negative	AOAC
Storage	Store in cool & dry place. Do not freeze.<br>Keep away from strong light and heat.		
Shelf life	2 years when properly stored.		

*No.*3 *Malpighia emarginata* extract

**Product Name**	*Malpighia emarginata* extract	
Lot Number	T0237	
Quantity	1500 kg	
Manufacture Date	2022-03-11	
Testing Date	2022-03-13	
**Analysis**	**Specification**	**Results**
Appearance	Light pink or red powder	Complies
Ash	≤ 5.0%	1.6%
Moisture	≤ 5.0%	3.85%
Odor	Characteristic	Complies
Particle size	100% through 80 mesh	Complies
Bulk Density	0.50-0.70 (g/ml)	Complies
**Microbiological**		
Total of bacteria	≤ 1000 cfu/g	Complies
Fungi	≤ 100 cfu/g	Complies
Salmonella	Negative	Complies
E. coli	Negative	Complies
Storage	Store in cool & dry place. Do not freeze.<br>Keep away from strong light and heat.	
Shelf life	2 years when properly stored.	

*No.*4 *Senna alexandrina* extract

<table>
<tr><td>Product Name</td><td colspan="3">Senna alexandrina extract</td></tr>
<tr><td>Lot Number</td><td>T0238</td><td>Manufacture Date</td><td>2021-09-12</td></tr>
<tr><td>Batch Quantity</td><td>800 kg</td><td>Expiry Date</td><td>2023-09-11</td></tr>
<tr><td>Part Used</td><td>Leaf</td><td>Extraction Solvent</td><td>Water & Ethanol</td></tr>
<tr><td>Analysis</td><td>Specification</td><td colspan="2">Results</td></tr>
<tr><td>Appearance</td><td>Brown powder</td><td>Complies</td><td>Organoleptic</td></tr>
<tr><td>Particle Size</td><td>95% through 80 mesh</td><td>Complies</td><td>USP<786></td></tr>
<tr><td>Bulk Density</td><td>30 - 65 g/100 mL</td><td>Complies</td><td>USP<616></td></tr>
<tr><td>Loss on Drying</td><td>≤ 5.00%</td><td>3.86%</td><td>USP<731></td></tr>
<tr><td>Ash</td><td>≤ 5.00%</td><td>1.62%</td><td>USP<281></td></tr>
<tr><td colspan="4">Contaminants</td></tr>
<tr><td>Lead (Pb)</td><td>≤ 3.0 ppm</td><td>Complies</td><td>ICP-MS||USP<730></td></tr>
<tr><td>Arsenic (As)</td><td>≤ 2.0 ppm</td><td>Complies</td><td>ICP-MS||USP<730></td></tr>
<tr><td>Cadmium (Cd）</td><td>≤ 1.0 ppm</td><td>Complies</td><td>ICP-MS||USP<730></td></tr>
<tr><td>Mercury (Hg)</td><td>≤ 0.1 ppm</td><td>Complies</td><td>ICP-MS||USP<730></td></tr>
<tr><td>Solvents Residue</td><td>Meet USP Requirements</td><td>Complies</td><td>GC||USP<467></td></tr>
<tr><td>Pesticides Residue</td><td>Meet USP Requirements</td><td>Complies</td><td>USP<561></td></tr>
<tr><td colspan="4">Microbiological</td></tr>
<tr><td>Total Plate Count</td><td>≤ 1000 cfu/g</td><td>Complies</td><td>USP<2021></td></tr>
<tr><td>Yeast & Mold</td><td>≤ 100 cfu/g</td><td>Complies</td><td>USP<2021></td></tr>
<tr><td>E. coli</td><td>Absent</td><td>Complies</td><td>USP<2022></td></tr>
<tr><td>Salmonella</td><td>Absent</td><td>Complies</td><td>USP<2022></td></tr>
<tr><td>Staphylococcus aureus</td><td>Absent</td><td>Complies</td><td>USP<2022></td></tr>
<tr><td colspan="4">Storage Packed in a polyethylene lined corrugated package
Stored in a well-closed container away from light, heat and moisture
Net Weight: 25 kg/drum.
Shelf life 2 years if sealed and stored properly.</td></tr>
</table>

*No.*5 *Nelumbinis folium* extract

**Product Name**	*Nelumbinis folium* extract	
Lot Number	T0239	
Quantity	350 kg	
Manufacture Date	2022-05-13	
Date of Test	2022-05-16	
**Analysis**	**Specification**	**Results**
Appearance	Brown fine powder	Complies
Ash	≤ 5.0%	2.5%
Moisture	≤ 6.0%	4.6%
Pesticides	≤ 2 ppm	Complies
Heavy metals	≤ 10 ppm	Complies
Odor	Characteristic	Complies
Particle size	100% through 80 mesh	Complies
**Microbiological**		
Total of bacteria	≤ 10000 cfu/g	Complies
Fungi	≤ 100 cfu/g	Complies
Salmonella	Negative	Complies
E. coli	Negative	Complies
Storage	Store in cool & dry place. Do not freeze.<br>Keep away from strong light and heat.	
Shelf life	2 years when properly stored.	

No.6 *Momordica charantia* extract

**Product Name**	*Momordica charantia* extract		
Batch Number	T0240		
Manufacture Date	2021-10-25	Expiry Date	2021-10-26
**Analysis**	**Specification**	**Results**	
Appearance	Brown powder	Complies	*Organoleptic*
Particle Size	100% through 80 mesh	Complies	CP2015
Bulk Density	40 - 60 g/100 mL	52.5 g/100 mL	CP2015
Loss on Drying	≤ 5.00%	3.39%	GB 5009.3
Ash	≤ 8.00%	6.18%	GB 5009.4
**Contaminants**			
Lead(Pb)	≤ 1.5 ppm	Complies	GB 5009.12
Arsenic(As)	≤ 1.0 ppm	Complies	GB 5009.11
Cadmium(Cd）	≤ 0.3 ppm	Complies	GB 5009.15
Mercury(Hg)	≤ 0.3 ppm	Complies	GB 5009.17
**Microbiological**			
Total Plate Count	≤ 1000 cfu/g	Complies	GB 4789.2
Molds	≤ 50 cfu/g	Complies	GB 4789.15
E. coli	Absent	Complies	GB 4789.3
Salmonella	Absent	Complies	GB 4789.4
Staphylococcus aureus	Absent	Complies	GB 4789.10
Storage Store in cool & dry place. Do not freeze. Keep away from strong light and heat.<br>Shelf life 2 years when properly stored.			

No.7 Aloe vera extract

**Product Name**	*Aloe vera* extract	
Lot Number	T0241	
Manufacture Date	2021-10-21	
Expiry Date	2021-10-23	
**Analysis**	**Specification**	**Results**
Appearance	Brown powder	Complies
Particle Size	100% through 80 mesh	Complies
Moisture	≤ 5.00%	2.6%
Ash	≤ 5.00%	1.9%
**Contaminants**		
Heavy metals	≤ 10 ppm	Complies
Lead (Pb)	≤ 2.0 ppm	Complies
Arsenic (As)	≤ 2.0 ppm	Complies
**Microbiological**		
Total Plate Count	≤ 1000 cfu/g	Complies
Molds	≤ 100 cfu/g	Complies
E. coli	Negative	Complies
Salmonella	Negative	Complies
Storage Store in cool & dry place. Do not freeze.<br>Keep away from strong light and heat.<br>Shelf life 2 years when properly stored.		

*No.*8 *Gentiana scabra* extract

**Product Name**	Gentiana scabra extract	Lot Number	T0242
Manufacture Date	2021-05-02	Analysis Date	2021-05-03
Quantity	1000 kg	Certificate Date	2021-05-10
**Analysis**	**Specification**	**Results**	
Appearance	Fine powder	Organoleptic	Complies
Color	Yellow brown	Organoleptic	Complies
Odor＆Taste	Characteristic	Organoleptic	Complies
Loss On Drying	≤ 5.0%	Eur.Ph.6.0<2.8.17>	4.35%
Ash	≤ 5.0%	Eur.Ph.6.0<2.4.16>	4.32%
Sieve Analysis	98% pass 80 mesh	USP34<786>	Complies
Heavy Metals	≤ 20 ppm	Eur.Ph.7.0<2.2.58>	9 ppm
Cadmium (Cd)	≤ 1 ppm	Eur.Ph.7.0<2.2.58>	Complies
Mercury (Hg)	≤ 0.1 ppm	Eur.Ph.7.0<2.2.58>	Complies
Arsenic (As)	≤ 1 ppm	Eur.Ph.7.0<2.2.58>	Complies
Lead (Pb)	≤ 3 ppm	Eur.Ph.7.0<2.2.58>	Complies
Bulk density	45 - 65 g/100 mL	Eur.Ph.7.0<2.9.15>	Complies
**Microbiological**			
Total plate count	≤1000 cfu/g	USP34<61>	Complies
Yeast & mold	≤100 cfu/g	USP34<61>	Complies
E. coli	Negative	USP34<62>	Negative
Salmonella	Negative	USP34<62>	Negative
Storage	Store in cool & dry place. Do not freeze. Keep away from strong light and heat.		
Shelf life	2 years when properly stored.		

No.9 Lycium chinense extract

**Product Name**	*Lycium chinense* extract	
Lot Number	T0243	
Manufacture Date	2020-12-23	
Expiry Date	2020-12-24	
**Analysis**	**Specification**	**Results**
Appearance	Red-brown powder	Complies
Particle Size	100% through 80 mesh	Complies
Ash	≤ 5.0%	4.0%
Moisture	≤ 8.0%	3.85%
**Contaminants**		
Heavy metals	≤ 10 ppm	Complies
Pesticides	Negative	Complies
**Microbiological**		
Total Plate Count	≤ 1000 cfu/g	Complies
Molds	≤ 100 cfu/g	Complies
E. coli	Negative	Complies
Salmonella	Negative	Complies
Storage Store in cool & dry place. Do not freeze. Keep away from strong light and heat. Shelf life 2 years when properly stored.		

*No.*10 *Helianthus tuberosus* extract

**Product Name**	*Helianthus tuberosus* extract	
Lot Number	T0244	
Manufacture Date	2022-03-11	
Expiry Date	2022-03-13	
**Analysis**	**Specification**	**Results**
Appearance	White fine powder	Complies
Particle Size	95% through 80 mesh	Complies
Loss on Drying	≤ 5.00%	3.20%
pH	4.5 - 7.0	3.2
**Contaminants**		
Heavy metals	≤ 20 ppm	Complies
As	< 2 ppm	Complies
**Microbiological**		
Total Plate Count	≤ 1000 cfu/g	260 cfu/g
Molds	≤ 100 cfu/g	20 cfu/g
E. coli	Negative	Complies
Salmonella	Negative	Complies
Storage	Store in cool & dry place. Do not freeze. Keep away from strong light and heat.	
Shelf life	2 years when properly stored.	

*No.*11 *Folium mori* extract

**Product Name**	*Folium mori* extract	
Batch Number	T0246	
Manufacture Date	2022-04-21	
Expiry Date	2022-04-23	
**Analysis**	**Specification**	**Results**
Appearance	Brown powder	Complies
Particle Size	95% through 80 mesh	Complies
Loss on Drying	≤ 5.00%	4.06%
AshContent	≤ 8.0%	5.13%
**Contaminants**		
Pb	≤ 2 ppm	Complies
As	≤ 1 ppm	Complies
Hg	≤ 0.3 ppm	Complies
**Microbiological**		
Total Plate Count	≤ 1000 cfu/g	Complies
Molds	≤ 100 cfu/g	Complies
E. coli	Negative	Complies
Salmonella	Negative	Complies
Staph. aureus	Negative	Complies
Storage Store in cool & dry place. Do not freeze. Keep away from strong light and heat.<br>Shelf life 2 years when properly stored.		

*No.*12 *Trifolium pratense* extract

**Product Name**	*Trifolium pratense* extract	
Lot Number	T0247	
Quantity	200 kg	
Manufacture Date	2022-05-11	
Testing Date	2022-05-13	
**Analysis**	**Specification**	**Results**
Appearance	Brown-black fine powder	Complies
Ash	≤ 5.0%	3.5%
Moisture	≤ 5.0%	3.02%
Heavy metals	≤ 10 ppm	Complies
Pb	≤ 2.0 ppm	Complies
As	≤ 2.0 ppm	Complies
Hg	≤ 1.0 ppm	Complies
Cd	≤ 1.0 ppm	Complies
Odor	Characteristic	Complies
Particle size	100% through 80 mesh	Complies
**Microbiological**		
Total of bacteria	≤ 1000 cfu/g	Complies
Fungi	≤ 100 cfu/g	Complies
Salmonella	Negative	Complies
E. coli	Negative	Complies
Storage	Store in cool & dry place. Do not freeze. Keep away from strong light and heat.	
Shelf life	2 years when properly stored.	

*No.*13 *Serenoa repens* extract

**Product Name**	*Serenoa repens* extract	
Lot Number	T0248	
Quantity	2000 kg	
Manufacture Date	2022-04-13	
Testing Date	2022-04-15	
**Analysis**	**Specification**	**Results**
Appearance	White fine powder	Complies
Ash	≤ 5.0%	4.0%
Moisture	≤ 5.0%	3.2%
Heavy metals	≤ 15 ppm	Complies
Pb	≤ 1.0 ppm	Complies
As	≤ 2.0 ppm	Complies
Hg	≤ 0.2 ppm	Complies
Odor	Characteristic	Complies
Particle size	100% through 80 mesh	Complies
**Microbiological**		
Total of bacteria	≤ 3000 cfu/g	Complies
Fungi	≤ 100 cfu/g	Complies
Salmonella	Negative	Complies
E. coli	Negative	Complies
Storage	Store in cool & dry place. Do not freeze.<br>Keep away from strong light and heat.	
Shelf life	2 years when properly stored.	

*No.*14 *Olea europaea L* extract

**Product Name**	*Olea europaea L* extract	
Lot Number	DG0132	
Quantity	1500 kg	
Manufacture Date	2021-12-05	
**Analysis**	**Specification**	**Results**
Appearance	Brown powder	Complies
Loss on Drying	≤ 5.0%	3.72%
Ash	≤ 5.0%	2.95%
Heavy metals	≤ 10 ppm	Complies
Pb	≤ 2 ppm	Complies
As	≤ 1 ppm	Complies
Hg	≤ 0.1 ppm	Complies
Cd	≤ 1 ppm	Complies
Odor	Characteristic	Complies
Particle size	95% through 80 mesh	Complies
**Microbiological**		
Total of bacteria	≤ 1000 cfu/g	Complies
Fungi	≤ 100 cfu/g	Complies
Salmonella	Negative	Complies
E. coli	Negative	Complies
Storage	Store in cool & dry place. Do not freeze. Keep away from strong light and heat.	
Shelf life	1 year when properly stored.	

*No.*15 *Eleutherococcus senticosus* extract

**Product Name**	*Eleutherococcus senticosus* extract	
Lot Number	T0061	
Manufacture Date	2022-01-09	
Testing Date	2022-01-11	
**Analysis**	**Specification**	**Results**
Assay	≥ 0.8%	0.83%
Appearance	Brown-yellow fine powder	Complies
Ash	≤ 5.0%	3.15%
Moisture	≤ 5.0%	3.0%
Heavy metals	≤ 10 ppm	Complies
Pb	≤ 2.0 ppm	Complies
As	≤ 2.0 ppm	Complies
Hg	≤ 0.5 ppm	Complies
Odor	Characteristic	Complies
Particle size	100% through 80 mesh	Complies
**Microbiological**		
Total of bacteria	≤ 10000 cfu/g	Complies
Fungi	≤ 1000 cfu/g	Complies
Salmonella	Negative	Complies
E. coli	Negative	Complies
Storage Store in cool & dry place. Do not freeze.<br>Keep away from strong light and heat.<br>Shelf life 2 years when properly stored.		

*No.*16 *Yucca smalliana* extract

**Product Name**	*Yucca smalliana* extract	
Part Used	Stems and leaves	
Extract solvent	Ethanol & Water	
Lot Number	T0253	
Quantity	500 kg	
Manufacture Date	2022-05-16	
Testing Date	2022-05-18	
**Analysis**	**Specification**	**Results**
Appearance	Brown fine powder	Complies
Ash	≤ 5.0%	3.06%
Moisture	≤ 5.0%	3.15%
Heavy metals	≤ 20 ppm	Complies
Pb	≤ 2.0 ppm	Complies
As	≤ 2.0 ppm	Complies
Odor	Characteristic	Complies
Particle size	100% through 80 mesh	Complies
**Microbiological**		
Total of bacteria	≤ 1000 cfu/g	Complies
Fungi	≤ 100 cfu/g	Complies
Salmonella	Negative	Complies
E. coli	Negative	Complies
Storage	Store in cool & dry place, don't freeze. Keep away from strong light and heat.	
Shelf life	2 years when properly stored.	

*No.*17 *Valeriana officinalis* extract

**Product Name**	*Valeriana officinalis* extract	
Lot Number	T0256	
Manufacture Date	2022-05-07	
Testing Date	2022-05-09	
**Analysis**	**Specification**	**Results**
Appearance	Dark brown powder	Complies
Ash	≤ 5.0%	2.3%
Moisture	≤ 5.0%	3.6%
Pesticides	Negative	Complies
Heavy metals	≤ 10 ppm	Complies
Pb	≤ 2.0 ppm	Complies
As	≤ 2.0 ppm	Complies
Odor	Characteristic	Complies
Particle size	100% through 80 mesh	Complies
**Microbiological**		
Total of bacteria	≤ 1000 cfu/g	Complies
Fungi	≤ 100 cfu/g	Complies
Salmonella	Negative	Complies
E. coli	Negative	Complies
Storage	Store in cool & dry place, don't freeze.<br>Keep away from strong light and heat.	
Shelf life	2 years when properly stored.	

*No.*18 *Oryza sativa f. nigrescens* extract

**Product Name**	*Oryza sativa f. nigrescens* extract	
Lot Number	T0249	
Quantity	820 kg	
Manufacture Date	2022-06-11	
Date of Test	2022-06-14	
**Analysis**	**Specification**	**Results**
Appearance	Purple black fine powder	Complies
Excipient	None	Complies
Ash	≤ 5.0%	1.65%
Moisture	≤ 5.0%	3.74%
Heavy metals	≤ 10 ppm	Complies
Pb	≤ 1.0 ppm	≤ 0.1 ppm
As	≤ 1.0 ppm	≤ 0.2 ppm
Hg	≤ 0.1 ppm	≤ 0.02 ppm
Cd	≤ 1.0 ppm	≤ 0.1 ppm
Odor	Characteristic	Complies
Particle size	100% through 80 mesh	Complies
**Microbiological**		
Total of bacteria	≤ 1000 cfu/g	Complies
Fungi	≤ 100 cfu/g	Complies
Salmonella	Negative	Complies
E. coli	Negative	Complies
Storage	Store in cool & dry place. Do not freeze.<br>Keep away from strong light and heat.	
Shelf life	2 years when properly stored.	

*No.*19 *Garcinia cambogia* extract

**Product Name**	*Garcinia cambogia* extract	
Lot Number	T0258	
Manufacture Date	2022-02-23	
**Analysis**	**Specification**	**Results**
Appearance	Fine powder	Complies
Color	Off-white to creamy	Complies
Odor	Characteristic	Complies
Taste	Characteristic	Complies
Particle Size	95% through 80 mesh	Complies
Loss on Drying	≤ 5.0%	4.07%
Ash	≤ 5.0%	3.12%
Loose Density	0.30 - 0.60 g/mL	0.29 g/mL
Tight Density	0.40 - 0.80 g/mL	0.45 g/mL
Lead (Pb)	≤ 2.0 ppm	Complies
Arsenic (As)	≤ 1.0 ppm	Complies
Mercury (Hg)	≤ 0.1 ppm	Complies
Cadmium (Cd)	≤ 0.3 ppm	Complies
**Microbiological**		
Total Plate Count	≤ 1000 cfu/g	Complies
Yeast & Mold	≤ 100 cfu/g	Complies
E. coli	Negative	Complies
Salmonella	Negative	Complies
Storage	Store in cool & dry place. Do not freeze.<br>Keep away from strong light and heat.	
Shelf life	2 years when properly stored.	

*No.*20 *Coffea arabica* extract

**Product Name**	*Coffea arabica* extract	
Lot Number	T0267	
Quantity	1200 kg	
Manufacture Date	2021-12-10	
Date of Test	2021-12-12	
**Analysis**	**Specification**	**Results**
Appearance	Brown powder	Complies
Moisture	≤ 5.0%	4.56%
Ash	≤ 5.0%	4.18%
Total Heavy Metals	< 10 ppm	Complies
Moisture	≤ 5.0%	3.74%
Heavy metals	≤ 10 ppm	Complies
Pb	≤ 1.5 ppm	Complies
As	≤ 1.0 ppm	Complies
Hg	≤ 0.3 ppm	Complies
Cd	≤ 0.3 ppm	Complies
Odor	Unique smell	Complies
Taste	Unique taste	Complies
Particle size	100% through 80 mesh	Complies
**Microbiological**		
Total of bacteria	≤ 1000 cfu/g	Complies
Fungi	≤ 50 cfu/g	Complies
Salmonella	Negative	Complies
E. coli	Negative	Complies
Storage	Store in cool & dry place. Do not freeze.<br>Keep away from strong light and heat.	
Shelf life	2 years when properly stored.	

*No.*21 *Sapindus saponaria* extract

**Product Name**	*Sapindus saponaria* extract	
Lot Number	T0250	
Quantity	800 kg	
Manufacture Date	2022-05-19	
Testing Date	2022-05-21	
**Analysis**	**Specification**	**Results**
Appearance	Light yellow fine powder	Complies
Ash	≤ 5.0%	1.35%
Moisture	≤ 5.0%	2.46%
Heavy metals	≤ 10 ppm	Complies
Pb	≤ 0.5 ppm	Complies
As	≤ 0.5 ppm	Complies
Hg	≤ 0.02 ppm	Complies
Cd	≤ 0.05 ppm	Complies
Odor	Characteristic	Complies
Particle size	100% through 80 mesh	Complies
**Microbiological**		
Total of bacteria	≤ 1000 cfu/g	Complies
Fungi	≤ 100 cfu/g	Complies
Salmonella	Negative	Complies
E. coli	Negative	Complies
Storage: Store in cool & dry place. Do not freeze. Keep away from strong light and heat.<br>Shelf life: 2 years when properly stored.		

*No.*22 *Echinacea purpurea* extract

**Product Name**	*Echinacea purpurea* extract	
Lot Number	T0262	
Manufacture Date	2022-01-19	
Testing Date	2022-01-21	
**Analysis**	**Specification**	**Results**
Appearance	Brown fine powder	Complies
Ash	≤ 5.0%	3.2%
Moisture	≤ 5.0%	3.5%
Pesticides	Negative	Complies
Heavy metals	≤ 10 ppm	Complies
Pb	≤ 2.0 ppm	Complies
As	≤ 2.0 ppm	Complies
Odor	Characteristic	Complies
Particle size	100% through 80 mesh	Complies
**Microbiological**		
Total of bacteria	≤ 3000 cfu/g	Complies
Fungi	≤ 100 cfu/g	Complies
Salmonella	Negative	Complies
E. coli	Negative	Complies
Storage	Store in cool & dry place. Do not freeze.<br>Keep away from strong light and heat.	
Shelf life	2 years when properly stored.	

*No.*23 *Helleborus thibetanus* extract

**Product Name**	*Helleborus thibetanus* extract	
Lot Number	DT0221	
Quantity	200 kg	
Manufacture Date	2022-04-16	
Testing Date	2022-04-21	
**Analysis**	**Specification**	**Results**
Appearance	Brown powder	Complies
Ash	≤ 2.0%	0.8%
Moisture	≤ 5.0%	1.2%
666	< 0.2 ppm	Complies
DDT	< 0.2 ppm	Complies
Heavy metals	≤ 10 ppm	Complies
Pb	≤ 2.0 ppm	Complies
As	≤ 2.0 ppm	Complies
Odor	Characteristic	Complies
Particle size	100% through 80 mesh	Complies
**Microbiological**		
Total of bacteria	≤ 1000 cfu/g	Complies
Fungi	≤ 100 cfu/g	Complies
Salmonella	Negative	Complies
E. coli	Negative	Complies
Storage Store in cool & dry place. Do not freeze.<br>Keep away from strong light and heat.<br>Shelf life 2 years when properly stored.		

*No.*24 *Garcinia mangostana* extract

**Product Name**	*Garcinia mangostana* extract	
Lot Number	T0266	
Quantity	800 kg	
Manufacture Date	2022-05-14	
Testing Date	2022-05-16	
**Analysis**	**Specification**	**Results**
Appearance	Brown-yellow powder	Complies
Ash	≤ 10.0%	7.3%
Moisture	≤ 5.0%	4.2%
Heavy metals	≤ 10 ppm	Complies
Pb	≤ 0.5 ppm	Complies
As	≤ 0.5 ppm	Complies
Hg	≤ 0.1 ppm	Complies
Cd	≤ 0.05 ppm	Complies
Odor	Characteristic	Complies
Particle size	100% through 80 mesh	Complies
666	≤ 0.1 ppm	Complies
DDT	≤ 0.1 ppm	Complies
Acephate	≤ 0.1 ppm	Complies
Methamidophos	≤ 0.1 ppm	Complies
Parathion	≤ 0. 1ppm	Complies
Pcnb	≤ 10 ppb	Complies
**Microbiological**		
Total of bacteria	≤ 1000 cfu/g	Complies
Fungi	≤ 100 cfu/g	Complies
Salmonella	Negative	Complies
E. coli	Negative	Complies
Staphylococcus	Negative	Complies
Storage	Store in cool & dry place. Do not freeze.<br>Keep away from strong light and heat.	
Shelf life	2 years when properly stored.	

No.25 *Eriobotrya japonica* extract

**Product Name**	*Eriobotrya japonica* extract	
Lot Number	T0251	
Quantity	300 kg	
Manufacture Date	2022-04-20	
Testing Date	2022-04-21	
**Analysis**	**Specification**	**Results**
Appearance	Yellow-green powder	Complies
Ash	≤ 5.0%	2.3%
Moisture	≤ 5.0%	3.6%
Pesticides	Negative	Complies
Heavy metals	≤ 10 ppm	Complies
Pb	≤ 2.0 ppm	Complies
As	≤ 2.0 ppm	Complies
Odor	Characteristic	Complies
Particle size	100% through 80 mesh	Complies
Solubility	Slightly soluble in ethanol	Complies
**Microbiological**		
Total of bacteria	≤ 1000 cfu/g	Complies
Fungi	≤ 100 cfu/g	Complies
Salmonella	Negative	Complies
E. coli	Negative	Complies
Storage	Store in cool & dry place. Do not freeze.<br>Keep away from strong light and heat.	
Shelf life	2 years when properly stored.	

*No.*26 *Silybum marianum* extract

**Product Name**	*Silybum marianum* extract	
Lot Number	T0255	
Quantity	1100 kg	
Manufacture Date	2022-03-16	
Testing Date	2022-03-18	
**Analysis**	**Specification**	**Results**
Appearance	Light yellow powder	Complies
Ash	≤ 1.0%	0.35%
Moisture	≤ 5.0%	2.82%
Solvent Residue	≤ 1.0%	Complies
Heavy metals	≤ 20 ppm	Complies
Pb	≤ 2.0 ppm	Complies
As	≤ 2.0 ppm	Complies
Odor	Characteristic	Complies
Particle size	100% through 80 mesh	Complies
**Microbiological**		
Total of bacteria	≤ 1000 cfu/g	Complies
Fungi	≤ 100 cfu/g	Complies
Salmonella	Negative	Complies
E. coli	Negative	Complies
Storage Store in cool & dry place. Do not freeze.<br>Keep away from strong light and heat.<br>Shelf life 2 years when properly stored.		

*No.*27 *Centella asiatica* extract

**Product Name**	*Centella asiatica* extract	
Lot Number	T0227	
Quantity	380 kg	
Manufacture Date	2022-04-11	
Testing Date	2022-04-12	
**Analysis**	**Specification**	**Results**
Appearance	Yellow fine powder	Complies
Ash	≤ 5.0%	3.06%
Moisture	≤ 5.0%	4.15%
Pesticides	Negative	Complies
Heavy metals	≤ 10 ppm	Complies
Pb	≤ 5.0 ppm	Complies
As	≤ 2.0 ppm	Complies
Hg	≤ 0.2 ppm	Complies
Cd	≤ 0.3 ppm	Complies
BHC	< 0.1 ppm	Complies
DDT	< 0.1 ppm	Complies
PCNB	< 0.1 ppm	Complies
Aldrin	< 0.02 ppm	Complies
Odor	Characteristic	Complies
Particle size	100% through 80 mesh	Complies
**Microbiological**		
Total of bacteria	≤ 1000 cfu/g	Complies
Fungi	≤ 100 cfu/g	Complies
Salmonella	Negative	Complies
E. coli	Negative	Complies
Storage	Store in cool & dry place. Do not freeze. Keep away from strong light and heat.	
Shelf life	2 years when properly stored.	

*No.*28 *Salix babylonica* extract

**Product Name**	*Salix babylonica* extract	
Lot Number	T0261	
Quantity	1500 kg	
Manufacture Date	2022-03-12	
Testing Date	2022-03-14	
**Analysis**	**Specification**	**Results**
Appearance	Brown powder	Complies
Ash	≤ 20.0%	17.3%
Moisture	≤ 5.0%	3.5%
Heavy metals	≤ 10 ppm	Complies
Pb	≤ 2.0 ppm	Complies
As	≤ 2.0 ppm	Complies
Hg	≤ 1.0 ppm	Complies
Odor	Characteristic	Complies
Particle size	100% through 80 mesh	Complies
**Microbiological**		
Total of bacteria	≤ 1000 cfu/g	Complies
Fungi	≤ 100 cfu/g	Complies
Salmonella	Negative	Complies
E. coli	Negative	Complies
Storage	Store in cool & dry place. Do not freeze. Keep away from strong light and heat.	
Shelf life	2 years when properly stored.	

*No.*29 *Tagetes erecta* extract

**Product Name**	*Tagetes erecta* extract	
Lot Number	T0259	
Quantity	800 kg	
Manufacture Date	2022-02-19	
Testing Date	2022-02-20	
**Analysis**	**Specification**	**Results**
Appearance	Yellow-orange fine powder	Complies
Ash	≤ 5.0%	2.3%
Moisture	≤ 5.0%	1.2%
Pesticides	Negative	Complies
Heavy metals	≤ 10 ppm	Complies
Pb	≤ 1.0 ppm	Complies
As	≤ 1.0 ppm	Complies
Hg	≤ 0.2 ppm	Complies
Odor	Characteristic	Complies
Particle size	100% through 80 mesh	Complies
**Microbiological**		
Total of bacteria	≤ 1000 cfu/g	Complies
Fungi	≤ 100 cfu/g	Complies
Salmonella	Negative	Complies
E. coli	Negative	Complies
Storage	Store in cool & dry place. Do not freeze.<br>Keep away from strong light and heat.	
Shelf life	2 years when properly stored.	

*No.*30 *Arctium lappa* extract

**Product Name**	*Arctium lappa* extract	
Lot Number	T0264	
Manufacture Date	2022-04-28	
Testing Date	2022-04-28	
**Analysis**	**Specification**	**Results**
Appearance	Yellow brown powder	Complies
Ash	≤ 5.0%	4.83%
Moisture	≤ 5.0%	3.86%
Pesticides	Negative	Complies
Heavy metals	≤ 10 ppm	Complies
Pb	≤ 2.0 ppm	Complies
As	≤ 2.0 ppm	Complies
Odor	Characteristic	Complies
Particle size	100% through 80 mesh	Complies
**Microbiological**		
Total of bacteria	≤ 1000 cfu/g	Complies
Fungi	≤ 100 cfu/g	Complies
Salmonella	Negative	Complies
E. coli	Negative	Complies
Storage	Store in cool & dry place. Do not freeze.<br>Keep away from strong light and heat.	
Shelf life	2 years when properly stored.	

*No.*31 *Paeonia lactiflora* extract

**Product Name**	*Paeonia lactiflora* extract	
Lot Number	T0265	
Quantity	500 kg	
Manufacture Date	2022-05-06	
Testing Date	2022-05-06	
**Analysis**	**Specification**	**Results**
Appearance	Brown powder	Complies
Ash	≤ 5.0%	4.23%
Moisture	≤ 5.0%	3.46%
Pesticides	Negative	Complies
Heavy metals	≤ 10 ppm	Complies
Pb	≤ 2.0 ppm	Complies
As	≤ 2.0 ppm	Complies
Odor	Characteristic	Complies
Particle size	100% through 80 mesh	Complies
**Microbiological**		
Total of bacteria	≤ 1000 cfu/g	Complies
Fungi	≤ 100 cfu/g	Complies
Salmonella	Negative	Complies
E. coli	Negative	Complies
Storage	Store in cool & dry place. Do not freeze. Keep away from strong light and heat.	
Shelf life	2 years when properly stored.	

*No.*32 *Rosmarinus officinalis* extract

**Product Name**	*Rosmarinus officinalis* extract	
Lot Number	DM0244	
Quantity	500 kg	
Manufacture Date	2022-02-18	
Testing Date	2022-02-20	
**Analysis**	**Specification**	**Results**
Appearance	Yellowish brown powder	Complies
Ash	≤ 7.0%	1.83%
Moisture	≤ 5.0%	3.86%
Heavy metals	≤ 10 ppm	Complies
Pb	≤ 2.0 ppm	Complies
As	≤ 2.0 ppm	Complies
Odor	Characteristic	Complies
Particle size	100% through 80 mesh	Complies
**Microbiological**		
Total of bacteria	≤ 1000 cfu/g	Complies
Fungi	≤ 100 cfu/g	Complies
Salmonella	Negative	Complies
E. coli	Negative	Complies
Storage Store in cool & dry place. Do not freeze.<br>Keep away from strong light and heat.<br>Shelf life 2 years when properly stored.		

*No.*33 *Lampranthus spectabilis* extract

**Product Name**	*Lampranthus spectabilis* extract	
Lot Number	T0268	
Quantity	1160 kg	
Manufacture Date	2022-04-24	
Testing Date	2022-04-26	
**Analysis**	**Specification**	**Results**
Appearance	Brown powder	Complies
Ash	≤ 10.0%	3.95%
Moisture	≤ 7.0%	4.67%
Heavy metals	≤ 10 ppm	Complies
Odor	Characteristic	Complies
Particle size	100% through 80 mesh	Complies
**Microbiological**		
Total of bacteria	≤ 1000 cfu/g	Complies
Fungi	≤ 100 cfu/g	Complies
Salmonella	Negative	Complies
E. coli	Negative	Complies
Storage Store in cool & dry place. Do not freeze.<br>Keep away from strong light and heat.<br>Shelf life 2 years when properly stored.		

*No.*34 ***Salvia hispanica*** **extract**

**Product Name**	*Salvia hispanica* extract	
Lot Number	T0269	
Manufacture Date	2022-04-19	
Testing Date	2022-04-21	
**Analysis**	**Specification**	**Results**
Appearance	Yellow-brown powder	Complies
Ash	≤ 10.0%	4.25%
Moisture	≤ 7.0%	4.19%
Heavy metals	≤ 10 ppm	Complies
Odor	Characteristic	Complies
Particle size	100% through 80 mesh	Complies
**Microbiological**		
Total of bacteria	≤ 1000 cfu/g	Complies
Fungi	≤ 100 cfu/g	Complies
Salmonella	Negative	Complies
E. coli	Negative	Complies
Storage Store in cool & dry place. Do not freeze.<br>Keep away from strong light and heat.<br>Shelf life 2 years when properly stored.		

*No.*35 *Annona muricata* extract

**Product Name**	*Annona muricata* extract	
Lot Number	T0270	
Quantity	1500 kg	
Manufacture Date	2022-06-15	
Testing Date	2022-06-18	
**Analysis**	**Specification**	**Results**
Appearance	Brown powder	Complies
Ash	≤ 10.0%	4.25%
Moisture	≤ 7.0%	3.37%
Heavy metals	≤ 10 ppm	Complies
Odor	Characteristic	Complies
Particle size	100% through 80 mesh	Complies
**Microbiological**		
Total of bacteria	≤ 1000 cfu/g	Complies
Fungi	≤ 100 cfu/g	Complies
Salmonella	Negative	Complies
E. coli	Negative	Complies
Storage	Store in cool & dry place. Do not freeze. Keep away from strong light and heat.	
Shelf life	2 years when properly stored.	

*No.*36 *Taraxacum mongolicum* extract

**Product Name**	*Taraxacum mongolicum* extract	
Lot Number	T0257	
Quantity	920 kg	
Manufacture Date	2022-03-16	
Testing Date	2022-03-18	
**Analysis**	**Specification**	**Results**
Appearance	Yellow-brown powder	Complies
Ash	≤ 10.0%	4.16%
Moisture	≤ 7.0%	4.12%
Heavy metals	≤ 10 ppm	Complies
Odor	Characteristic	Complies
Particle size	100% through 80 mesh	Complies
**Microbiological**		
Total of bacteria	≤ 1000 cfu/g	Complies
Fungi	≤ 100 cfu/g	Complies
Salmonella	Negative	Complies
E. coli	Negative	Complies
Storage	Store in cool & dry place. Do not freeze.<br>Keep away from strong light and heat.	
Shelf life	2 years when properly stored.	

*No.*37 *Polygonatum kingianum* extract

**Product Name**	*Polygonatum kingianum* extract	
Lot Number	T0260	
Quantity	7800 kg	
Manufacture Date	2022-04-16	
Testing Date	2022-04-18	
**Analysis**	**Specification**	**Results**
Appearance	Yellowish brown powder	Complies
Ash	≤ 10.0%	4.75%
Moisture	≤ 7.0%	3.89%
Heavy metals	≤ 10 ppm	Complies
Odor	Characteristic	Complies
Particle size	100% through 80 mesh	Complies
**Microbiological**		
Total of bacteria	≤ 1000 cfu/g	Complies
Fungi	≤ 100 cfu/g	Complies
Salmonella	Negative	Complies
E. coli	Negative	Complies
Storage	Store in cool & dry place. Do not freeze.<br>Keep away from strong light and heat.	
Shelf life	2 years when properly stored.	

*No.*38 *Cynomorium songaricum* extract

**Product Name**	*Cynomorium songaricum* extract	
Lot Number	T0276	
Manufacture Date	2022-06-14	
Testing Date	2022-06-16	
**Analysis**	**Specification**	**Results**
Appearance	Yellow-brown powder	Complies
Ash	≤ 10.0%	4.82%
Moisture	≤ 7.0%	3.48%
Heavy metals	≤ 10 ppm	Complies
Odor	Characteristic	Complies
Particle size	100% through 80 mesh	Complies
**Microbiological**		
Total of bacteria	≤ 1000 cfu/g	Complies
Fungi	≤ 100 cfu/g	Complies
Salmonella	Negative	Complies
E. coli	Negative	Complies
Storage	Store in cool & dry place. Do not freeze.<br>Keep away from strong light and heat.	
Shelf life	2 years when properly stored.	

*No.*39 *Cistanche deserticola* extract

**Product Name**	*Cistanche deserticola* extract	
Lot Number	T0273	
Quantity	960 kg	
Manufacture Date	2022-05-08	
Testing Date	2022-05-11	
**Analysis**	**Specification**	**Results**
Appearance	Yellow-brown powder	Complies
Ash	≤ 10.0%	5.25%
Moisture	≤ 7.0%	4.79%
Heavy metals	≤ 10 ppm	Complies
Odor	Characteristic	Complies
Particle size	100% through 80 mesh	Complies
**Microbiological**		
Total of bacteria	≤ 1000 cfu/g	Complies
Fungi	≤ 100 cfu/g	Complies
Salmonella	Negative	Complies
E. coli	Negative	Complies
Storage	Store in cool & dry place. Do not freeze. Keep away from strong light and heat.	
Shelf life	2 years when properly stored.	

*No.*40 *Eurycoma longifolia* extract

**Product Name**	*Eurycoma longifolia* extract	
Lot Number	T0277	
Quantity	1240 kg	
Manufacture Date	2022-03-03	
Testing Date	2022-03-09	
**Analysis**	**Specification**	**Results**
Appearance	Yellow-brown powder	Complies
Ash	≤ 10.0%	4.81%
Moisture	≤ 7.0%	4.74%
Heavy metals	≤ 10 ppm	Complies
Odor	Characteristic	Complies
Particle size	100% through 80 mesh	Complies
**Microbiological**		
Total of bacteria	≤ 1000 cfu/g	Complies
Fungi	≤ 100 cfu/g	Complies
Salmonella	Negative	Complies
E. coli	Negative	Complies
Storage	Store in cool & dry place. Do not freeze.<br>Keep away from strong light and heat.	
Shelf life	2 years when properly stored.	

*No.*41 *Cuscuta australis* extract

**Product Name**	*Cuscuta australis* extract	
Lot Number	T0283	
Quantity	1340 kg	
Manufacture Date	2022-06-25	
Testing Date	2022-06-27	
**Analysis**	**Specification**	**Results**
Appearance	Brown-yellow powder	Complies
Ash	≤ 10.0%	4.65%
Moisture	≤ 7.0%	3.97%
Heavy metals	≤ 10 ppm	Complies
Odor	Characteristic	Complies
Particle size	100% through 80 mesh	Complies
**Microbiological**		
Total of bacteria	≤ 1000 cfu/g	Complies
Fungi	≤ 100 cfu/g	Complies
Salmonella	Negative	Complies
E. coli	Negative	Complies
Storage Store in cool & dry place. Do not freeze.<br>Keep away from strong light and heat.<br>Shelf life 2 years when properly stored.		

*No.*42 *Nelumbinis semen* extract

**Product Name**	*Nelumbinis semen* extract	
Lot Number	T0271	
Quantity	800 kg	
Manufacture Date	2022-06-22	
Testing Date	2022-06-25	
**Analysis**	**Specification**	**Results**
Appearance	Brown-yellow powder	Complies
Ash	≤ 10.0%	4.88%
Moisture	≤ 7.0%	4.17%
Heavy metals	≤ 10 ppm	Complies
Odor	Characteristic	Complies
Particle size	100% through 80 mesh	Complies
**Microbiological**		
Total of bacteria	≤ 1000 cfu/g	Complies
Fungi	≤ 100 cfu/g	Complies
Salmonella	Negative	Complies
E. coli	Negative	Complies
Storage	Store in cool & dry place. Do not freeze.<br>Keep away from strong light and heat.	
Shelf life	2 years when properly stored.	

*No.*43 *Chrysanthemum morifolium* extract

**Product Name**	*Chrysanthemum morifolium* extract	
Lot Number	T0285	
Quantity	550 kg	
Manufacture Date	2022-06-11	
Testing Date	2022-06-15	
**Analysis**	**Specification**	**Results**
Appearance	Brown-yellow fine powder	Complies
Ash	≤ 10.0%	4.11%
Moisture	≤ 7.0%	3.17%
Heavy metals	≤ 10 ppm	Complies
Odor	Characteristic	Complies
Particle size	100% through 80 mesh	Complies
**Microbiological**		
Total of bacteria	≤ 1000 cfu/g	Complies
Fungi	≤ 100 cfu/g	Complies
Salmonella	Negative	Complies
E. coli	Negative	Complies
Storage	Store in cool & dry place. Do not freeze. Keep away from strong light and heat.	
Shelf life	2 years when properly stored.	

*No.*44 ***Dendrobii caulis*** extract

**Product Name**	*Dendrobii caulis* extract	
Lot Number	T0293	
Quantity	690 kg	
Manufacture Date	2022-03-17	
Testing Date	2022-03-20	
**Analysis**	**Specification**	**Results**
Appearance	Brown-yellow fine powder	Complies
Ash	≤ 10.0%	3.85%
Moisture	≤ 7.0%	2.75%
Heavy metals	≤ 10 ppm	Complies
Odor	Characteristic	Complies
Particle size	100% through 80 mesh	Complies
**Microbiological**		
Total of bacteria	≤ 1000 cfu/g	Complies
Fungi	≤ 100 cfu/g	Complies
Salmonella	Negative	Complies
E. coli	Negative	Complies
Storage	Store in cool & dry place. Do not freeze.<br>Keep away from strong light and heat.	
Shelf life	2 years when properly stored.	

*No.*45 *Semen hoveniae* extract

**Product Name**	*Semen hoveniae* extract	
Lot Number	T0307	
Manufacture Date	2022-06-13	
Testing Date	2022-06-18	
**Analysis**	**Specification**	**Results**
Appearance	Brown-yellow fine powder	Complies
Ash	≤ 10.0%	4.88%
Moisture	≤ 7.0%	3.97%
Heavy metals	≤ 10 ppm	Complies
Odor	Characteristic	Complies
Particle size	100% through 80 mesh	Complies
**Microbiological**		
Total of bacteria	≤ 1000 cfu/g	Complies
Fungi	≤ 100 cfu/g	Complies
Salmonella	Negative	Complies
E. coli	Negative	Complies
Storage Store in cool & dry place. Do not freeze.<br>Keep away from strong light and heat.<br>Shelf life 2 years when properly stored.		

*No.*46 *Phyllanthus emblica* extract

**Product Name**	*Phyllanthus emblica* extract	
Lot Number	T0314	
Quantity	650 kg	
Manufacture Date	2022-04-25	
Testing Date	2022-04-26	
**Analysis**	**Specification**	**Results**
Appearance	Brown-yellow fine powder	Complies
Ash	≤ 10.0%	4.25%
Moisture	≤ 7.0%	3.66%
Heavy metals	≤ 10 ppm	Complies
Odor	Characteristic	Complies
Particle size	100% through 80 mesh	Complies
**Microbiological**		
Total of bacteria	≤ 1000 cfu/g	Complies
Fungi	≤ 100 cfu/g	Complies
Salmonella	Negative	Complies
E. coli	Negative	Complies
Storage	Store in cool & dry place. Do not freeze.<br>Keep away from strong light and heat.	
Shelf life	2 years when properly stored.	

*No.*47 *Wolfiporia cocos* extract

**Product Name**	*Wolfiporia cocos* extract	
Lot Number	T0274	
Quantity	960 kg	
Manufacture Date	2022-06-08	
Testing Date	2022-06-13	
**Analysis**	**Specification**	**Results**
Appearance	Off-white powder	Complies
Ash	≤ 10.0%	4.95%
Moisture	≤ 7.0%	3.98%
Heavy metals	≤ 10 ppm	Complies
Odor	Characteristic	Complies
Particle size	100% through 80 mesh	Complies
**Microbiological**		
Total of bacteria	≤ 1000 cfu/g	Complies
Fungi	≤ 100 cfu/g	Complies
Salmonella	Negative	Complies
E. coli	Negative	Complies
Storage	Store in cool & dry place. Do not freeze.<br>Keep away from strong light and heat.	
Shelf life	2 years when properly stored.	

*No.*48 *Protea cynaroides* extract

**Product Name**	*Protea cynaroides* extract	
Lot Number	DP0090	
Quantity	850 kg	
Manufacture Date	2022-06-09	
Testing Date	2022-06-11	
**Analysis**	**Specification**	**Results**
Appearance	Brown-yellow fine powder	Complies
Ash	≤ 10.0%	4.65%
Moisture	≤ 7.0%	4.66%
Heavy metals	≤ 10 ppm	Complies
Odor	Characteristic	Complies
Particle size	100% through 80 mesh	Complies
**Microbiological**		
Total of bacteria	≤ 1000 cfu/g	Complies
Fungi	≤ 100 cfu/g	Complies
Salmonella	Negative	Complies
E. coli	Negative	Complies
Storage	Store in cool & dry place. Do not freeze.<br>Keep away from strong light and heat.	
Shelf life	2 years when properly stored.	

*No.*49 *Cassia tora* extract

**Product Name**	*Cassia tora* extract	
Lot Number	T0234	
Quantity	1500 kg	
Manufacture Date	2022-02-16	
Testing Date	2022-02-18	
**Analysis**	**Specification**	**Results**
Appearance	Brown-yellow powder	Complies
Ash	≤ 10.0%	3.25%
Moisture	≤ 7.0%	2.7%
Heavy metals	≤ 10 ppm	Complies
Odor	Characteristic	Complies
Particle size	100% through 80 mesh	Complies
**Microbiological**		
Total of bacteria	≤ 1000 cfu/g	Complies
Fungi	≤ 100 cfu/g	Complies
Salmonella	Negative	Complies
E. coli	Negative	Complies
Storage	Store in cool & dry place. Do not freeze.<br>Keep away from strong light and heat.	
Shelf life	2 years when properly stored.	

*No.*50 *Salvia japonica* extract

**Product Name**	*Salvia japonica* extract	
Lot Number	T0294	
Manufacture Date	2022-02-16	
Testing Date	2022-02-18	
**Analysis**	**Specification**	**Results**
Appearance	Brown powder	Complies
Ash	≤ 10.0%	3.25%
Moisture	≤ 7.0%	2.7%
Heavy metals	≤ 10 ppm	Complies
Odor	Characteristic	Complies
Particle size	100% through 80 mesh	Complies
**Microbiological**		
Total of bacteria	≤ 1000 cfu/g	Complies
Fungi	≤ 100 cfu/g	Complies
Salmonella	Negative	Complies
E. coli	Negative	Complies
Storage	Store in cool & dry place. Do not freeze.<br>Keep away from strong light and heat.	
Shelf life	2 years when properly stored.	

*No.*51 *Fallopia multiflora* extract

**Product Name**	*Fallopia multiflora* extract	
Lot Number	T0338	
Quantity	2000 kg	
Manufacture Date	2022-03-23	
Testing Date	2022-03-26	
**Analysis**	**Specification**	**Results**
Appearance	Brown-yellow powder	Complies
Ash	≤ 10.0%	4.13%
Moisture	≤ 7.0%	3.12%
Heavy metals	≤ 10 ppm	Complies
Odor	Characteristic	Complies
Particle size	100% through 80 mesh	Complies
**Microbiological**		
Total of bacteria	≤ 1000 cfu/g	Complies
Fungi	≤ 100 cfu/g	Complies
Salmonella	Negative	Complies
E. coli	Negative	Complies
Storage	Store in cool & dry place. Do not freeze. Keep away from strong light and heat.	
Shelf life	2 years when properly stored.	

*No.*52 *Opuntia stricta* extract

**Product Name**	*Opuntia stricta* extract	
Lot Number	T0339	
Quantity	1200 kg	
Manufacture Date	2022-04-16	
Testing Date	2022-04-18	
**Analysis**	**Specification**	**Results**
Appearance	Brown-yellow powder	Complies
Ash	≤ 10.0%	3.45%
Moisture	≤ 7.0%	2.71%
Heavy metals	≤ 10 ppm	Complies
Odor	Characteristic	Complies
Particle size	100% through 80 mesh	Complies
**Microbiological**		
Total of bacteria	≤ 1000 cfu/g	Complies
Fungi	≤ 100 cfu/g	Complies
Salmonella	Negative	Complies
E. coli	Negative	Complies
Storage	Store in cool & dry place. Do not freeze.<br>Keep away from strong light and heat.	
Shelf life	2 years when properly stored.	

*No.*53 *Leonurus artemisia* extract

**Product Name**	*Leonurus artemisia* extract	
Lot Number	T0315	
Manufacture Date	2022-03-26	
Testing Date	2022-03-29	
**Analysis**	**Specification**	**Results**
Appearance	Brown-yellow powder	Complies
Ash	≤ 10.0%	4.35%
Moisture	≤ 7.0%	2.51%
Heavy metals	≤ 10 ppm	Complies
Odor	Characteristic	Complies
Particle size	100% through 80 mesh	Complies
**Microbiological**		
Total of bacteria	≤ 1000 cfu/g	Complies
Fungi	≤ 100 cfu/g	Complies
Salmonella	Negative	Complies
E. coli	Negative	Complies
Storage	Store in cool & dry place. Do not freeze.<br>Keep away from strong light and heat.	
Shelf life	2 years when properly stored.	

*No.*54 ***Euphrasia pectinata*** **extract**

**Product Name**	*Euphrasia pectinata* extract	
Lot Number	T0340	
Quantity	950 kg	
Manufacture Date	2022-04-08	
Testing Date	2022-04-11	
**Analysis**	**Specification**	**Results**
Appearance	Brown-yellow powder	Complies
Ash	≤ 10.0%	3.47%
Moisture	≤ 7.0%	4.42%
Heavy metals	≤ 10 ppm	Complies
Odor	Characteristic	Complies
Particle size	100% through 80 mesh	Complies
**Microbiological**		
Total of bacteria	≤ 1000 cfu/g	Complies
Fungi	≤ 100 cfu/g	Complies
Salmonella	Negative	Complies
E. coli	Negative	Complies
Storage	Store in cool & dry place. Do not freeze.<br>Keep away from strong light and heat.	
Shelf life	2 years when properly stored.	

*No.*55 ***Plantago asiatica*** **extract**

**Product Name**	*Plantago asiatica* extract	
Lot Number	T0345	
Manufacture Date	2022-03-27	
Testing Date	2022-03-29	
**Analysis**	**Specification**	**Results**
Appearance	Brown-powder	Complies
Ash	≤ 10.0%	3.48%
Moisture	≤ 7.0%	3.96%
Heavy metals	≤ 10 ppm	Complies
Odor	Characteristic	Complies
Particle size	100% through 80 mesh	Complies
**Microbiological**		
Total of bacteria	≤ 1000 cfu/g	Complies
Fungi	≤ 100 cfu/g	Complies
Salmonella	Negative	Complies
E. coli	Negative	Complies
Storage	Store in cool & dry place. Do not freeze.<br>Keep away from strong light and heat.	
Shelf life	2 years when properly stored.	

*No.*56 *Asparagus cochinchinensis* extract

**Product Name**	*Asparagus cochinchinensis* extract	
Lot Number	T0284	
Manufacture Date	2022-03-29	
Testing Date	2022-03-31	
**Analysis**	**Specification**	**Results**
Appearance	Brown-yellow powder	Complies
Ash	≤ 10.0%	3.28%
Moisture	≤ 7.0%	3.95%
Heavy metals	≤ 10 ppm	Complies
Odor	Characteristic	Complies
Particle size	100% through 80 mesh	Complies
**Microbiological**		
Total of bacteria	≤ 1000 cfu/g	Complies
Fungi	≤ 100 cfu/g	Complies
Salmonella	Negative	Complies
E. coli	Negative	Complies
Storage	Store in cool & dry place. Do not freeze.<br>Keep away from strong light and heat.	
Shelf life	2 years when properly stored.	

*No.*57 *Vitex negundo* extract

**Product Name**	*Vitex negundo* extract	
Lot Number	T0327	
Quantity	970 kg	
Manufacture Date	2022-04-05	
Testing Date	2022-04-07	
**Analysis**	**Specification**	**Results**
Appearance	Brown powder	Complies
Ash	≤ 10.0%	3.31%
Moisture	≤ 7.0%	4.65%
Heavy metals	≤ 10 ppm	Complies
Odor	Characteristic	Complies
Particle size	100% through 80 mesh	Complies
**Microbiological**		
Total of bacteria	≤ 1000 cfu/g	Complies
Fungi	≤ 100 cfu/g	Complies
Salmonella	Negative	Complies
E. coli	Negative	Complies
Storage	Store in cool & dry place. Do not freeze. Keep away from strong light and heat.	
Shelf life	2 years when properly stored.	

*No.*58 *Morinda officinalis* extract

**Product Name**	*Morinda officinalis* extract	
Lot Number	T0321	
Quantity	1500 kg	
Manufacture Date	2022-04-10	
Testing Date	2022-04-12	
**Analysis**	**Specification**	**Results**
Appearance	Brown-yellow powder	Complies
Ash	≤ 10.0%	3.37%
Moisture	≤ 7.0%	2.69%
Heavy metals	≤ 10 ppm	Complies
Odor	Characteristic	Complies
Particle size	100% through 80 mesh	Complies
**Microbiological**		
Total of bacteria	≤ 1000 cfu/g	Complies
Fungi	≤ 100 cfu/g	Complies
Salmonella	Negative	Complies
E. coli	Negative	Complies
Storage	Store in cool & dry place. Do not freeze.<br>Keep away from strong light and heat.	
Shelf life	2 years when properly stored.	

*No.*59 *Cyperus rotundus* extract

**Product Name**	*Cyperus rotundus* extract	
Lot Number	T0341	
Quantity	1280 kg	
Manufacture Date	2022-04-19	
Testing Date	2022-04-21	
**Analysis**	**Specification**	**Results**
Appearance	Brown-yellow powder	Complies
Ash	≤ 10.0%	3.78%
Moisture	≤ 7.0%	2.56%
Heavy metals	≤ 10 ppm	Complies
Odor	Characteristic	Complies
Particle size	100% through 80 mesh	Complies
**Microbiological**		
Total of bacteria	≤ 1000 cfu/g	Complies
Fungi	≤ 100 cfu/g	Complies
Salmonella	Negative	Complies
E. coli	Negative	Complies
Storage	Store in cool & dry place. Do not freeze.<br>Keep away from strong light and heat.	
Shelf life	2 years when properly stored.	

*No.*60 *Houttuynia cordata* extract

**Product Name**	*Houttuynia cordata* extract	
Lot Number	T0083	
Quantity	1500 kg	
Manufacture Date	2022-04-12	
Testing Date	2022-04-15	
**Analysis**	**Specification**	**Results**
Appearance	Brown-yellow fine powder	Complies
Ash	≤ 10.0%	3.96%
Moisture	≤ 7.0%	2.73%
Heavy metals	≤ 10 ppm	Complies
Odor	Characteristic	Complies
Particle size	100% through 80 mesh	Complies
**Microbiological**		
Total of bacteria	≤ 1000 cfu/g	Complies
Fungi	≤ 100 cfu/g	Complies
Salmonella	Negative	Complies
E. coli	Negative	Complies
Storage	Store in cool & dry place. Do not freeze. Keep away from strong light and heat.	
Shelf life	2 years when properly stored.	

*No.*61 *Ajuga ciliata* extract

**Product Name**	*Ajuga ciliata* extract	
Lot Number	T0287	
Quantity	1300 kg	
Manufacture Date	2022-04-14	
Testing Date	2022-04-16	
**Analysis**	**Specification**	**Results**
Appearance	Brown powder	Complies
Ash	≤ 10.0%	4.06%
Moisture	≤ 7.0%	3.23%
Heavy metals	≤ 10 ppm	Complies
Odor	Characteristic	Complies
Particle size	100% through 80 mesh	Complies
**Microbiological**		
Total of bacteria	≤ 1000 cfu/g	Complies
Fungi	≤ 100 cfu/g	Complies
Salmonella	Negative	Complies
E. coli	Negative	Complies
Storage	Store in cool & dry place. Do not freeze.<br>Keep away from strong light and heat.	
Shelf life	2 years when properly stored.	

*No.*62 *Dictamnus dasycarpus* extract

**Product Name**	*Dictamnus dasycarpus* extract	
Lot Number	T0322	
Quantity	1400 kg	
Manufacture Date	2022-04-03	
Testing Date	2022-04-06	
**Analysis**	**Specification**	**Results**
Appearance	Brown powder	Complies
Ash	≤ 10.0%	3.25%
Moisture	≤ 7.0%	2.46%
Heavy metals	≤ 10 ppm	Complies
Odor	Characteristic	Complies
Particle size	100% through 80 mesh	Complies
**Microbiological**		
Total of bacteria	≤ 1000 cfu/g	Complies
Fungi	≤ 100 cfu/g	Complies
Salmonella	Negative	Complies
E. coli	Negative	Complies
Storage	Store in cool & dry place. Do not freeze.<br>Keep away from strong light and heat.	
Shelf life	2 years when properly stored.	

*No.*63 *Trigonella foenum-graecum* extract

**Product Name**	*Trigonella foenum-graecum* extract	
Lot Number	T0328	
Quantity	1300 kg	
Manufacture Date	2022-04-06	
Testing Date	2022-04-08	
**Analysis**	**Specification**	**Results**
Appearance	Yellow-brown powder	Complies
Ash	≤ 10.0%	3.41%
Moisture	≤ 7.0%	2.36%
Heavy metals	≤ 10 ppm	Complies
Odor	Characteristic	Complies
Particle size	100% through 80 mesh	Complies
**Microbiological**		
Total of bacteria	≤ 1000 cfu/g	Complies
Fungi	≤ 100 cfu/g	Complies
Salmonella	Negative	Complies
E. coli	Negative	Complies
Storage	Store in cool & dry place. Do not freeze.<br>Keep away from strong light and heat.	
Shelf life	2 years when properly stored.	

*No.*64 *Ficus carica* extract

**Product Name**	*Ficus carica* extract	
Lot Number	T0316	
Manufacture Date	2022-04-07	
Testing Date	2022-04-09	
**Analysis**	**Specification**	**Results**
Appearance	Off-white to white powder	Complies
Ash	≤ 10.0%	3.67%
Moisture	≤ 7.0%	2.46%
Heavy metals	≤ 10 ppm	Complies
Odor	Characteristic	Complies
Particle size	100% through 80 mesh	Complies
**Microbiological**		
Total of bacteria	≤ 1000 cfu/g	Complies
Fungi	≤ 100 cfu/g	Complies
Salmonella	Negative	Complies
E. coli	Negative	Complies
Storage Store in cool & dry place. Do not freeze.<br>Keep away from strong light and heat.<br>Shelf life 2 years when properly stored.		

*No.*65 *Alpinia oxyphylla* extract

**Product Name**	*Alpinia oxyphylla* extract	
Lot Number	T0317	
Quantity	1500 kg	
Manufacture Date	2022-04-13	
Testing Date	2022-04-15	
**Analysis**	**Specification**	**Results**
Appearance	Brown-yellow fine powder	Complies
Ash	≤ 10.0%	3.56%
Moisture	≤ 7.0%	2.13%
Heavy metals	≤ 10 ppm	Complies
Odor	Characteristic	Complies
Particle size	100% through 80 mesh	Complies
**Microbiological**		
Total of bacteria	≤ 1000 cfu/g	Complies
Fungi	≤ 100 cfu/g	Complies
Salmonella	Negative	Complies
E. coli	Negative	Complies
Storage	Store in cool & dry place. Do not freeze.<br>Keep away from strong light and heat.	
Shelf life	2 years when properly stored.	

*No.*66 *Ligusticum sinense* extract

**Product Name**	*Ligusticum sinense* extract	
Lot Number	T0346	
Manufacture Date	2022-04-14	
Testing Date	2022-04-16	
**Analysis**	**Specification**	**Results**
Appearance	Brown-yellow powder	Complies
Ash	≤ 10.0%	3.48%
Moisture	≤ 7.0%	2.23%
Heavy metals	≤ 10 ppm	Complies
Odor	Characteristic	Complies
Particle size	100% through 80 mesh	Complies
**Microbiological**		
Total of bacteria	≤ 1000 cfu/g	Complies
Fungi	≤ 100 cfu/g	Complies
Salmonella	Negative	Complies
E. coli	Negative	Complies
Storage	Store in cool & dry place. Do not freeze.<br>Keep away from strong light and heat.	
Shelf life	2 years when properly stored.	

*No.*67 ***Syzygium aromaticum*** **extract**

**Product Name**	*Syzygium aromaticum* extract	
Lot Number	T0278	
Quantity	1500 kg	
Manufacture Date	2022-04-15	
Testing Date	2022-04-17	
**Analysis**	**Specification**	**Results**
Appearance	Brown-yellow powder	Complies
Ash	≤ 10.0%	3.56%
Moisture	≤ 7.0%	2.09%
Heavy metals	≤ 10 ppm	Complies
Odor	Characteristic	Complies
Particle size	100% through 80 mesh	Complies
**Microbiological**		
Total of bacteria	≤ 1000 cfu/g	Complies
Fungi	≤ 100 cfu/g	Complies
Salmonella	Negative	Complies
E. coli	Negative	Complies
Storage	Store in cool & dry place. Do not freeze.<br>Keep away from strong light and heat.	
Shelf life	2 years when properly stored.	

*No.*68 *Aucklandiae radix* extract

**Product Name**	*Aucklandiae radix* extract	
Lot Number	T0263	
Quantity	1400 kg	
Manufacture Date	2022-04-21	
Testing Date	2022-04-23	
**Analysis**	**Specification**	**Results**
Appearance	Brown-yellow powder	Complies
Ash	≤ 10.0%	3.78%
Moisture	≤ 7.0%	2.64%
Heavy metals	≤ 10 ppm	Complies
Odor	Characteristic	Complies
Particle size	100% through 80 mesh	Complies
**Microbiological**		
Total of bacteria	≤ 1000 cfu/g	Complies
Fungi	≤ 100 cfu/g	Complies
Salmonella	Negative	Complies
E. coli	Negative	Complies
Storage	Store in cool & dry place. Do not freeze.<br>Keep away from strong light and heat.	
Shelf life	2 years when properly stored.	

*No.*69 *Angelica dahurica* extract

**Product Name**	*Angelica dahurica* extract	
Lot Number	T0323	
Quantity	1300 kg	
Manufacture Date	2022-04-20	
Testing Date	2022-04-22	
**Analysis**	**Specification**	**Results**
Appearance	Light brown powder	Complies
Ash	≤ 10.0%	3.56%
Moisture	≤ 7.0%	2.09%
Heavy metals	≤ 10 ppm	Complies
Odor	Characteristic	Complies
Particle size	100% through 80 mesh	Complies
**Microbiological**		
Total of bacteria	≤ 1000 cfu/g	Complies
Fungi	≤ 100 cfu/g	Complies
Salmonella	Negative	Complies
E. coli	Negative	Complies
Storage	Store in cool & dry place. Do not freeze.<br>Keep away from strong light and heat.	
Shelf life	2 years when properly stored.	

*No.*70 *Brassica oleracea var. italica* extract

**Product Name**	*Brassica oleracea var. italica* extract	
Lot Number	T0342	
Quantity	1300 kg	
Manufacture Date	2022-04-23	
Testing Date	2022-04-25	
**Analysis**	**Specification**	**Results**
Appearance	Brown-yellow powder	Complies
Ash	≤ 10.0%	3.45%
Moisture	≤ 7.0%	2.29%
Heavy metals	≤ 10 ppm	Complies
Odor	Characteristic	Complies
Particle size	100% through 80 mesh	Complies
**Microbiological**		
Total of bacteria	≤ 1000 cfu/g	Complies
Fungi	≤ 100 cfu/g	Complies
Salmonella	Negative	Complies
E. coli	Negative	Complies
Storage	Store in cool & dry place. Do not freeze. Keep away from strong light and heat.	
Shelf life	2 years when properly stored.	

*No.*71 *Alisma plantago-aquatica* extract

**Product Name**	*Alisma Plantago-aquatica* extract	
Lot Number	T0308	
Quantity	1400 kg	
Manufacture Date	2022-04-25	
Testing Date	2022-04-27	
**Analysis**	**Specification**	**Results**
Appearance	Brown-yellow powder	Complies
Ash	≤ 10.0%	3.56%
Moisture	≤ 7.0%	2.89%
Heavy metals	≤ 10 ppm	Complies
Odor	Characteristic	Complies
Particle size	100% through 80 mesh	Complies
**Microbiological**		
Total of bacteria	≤ 1000 cfu/g	Complies
Fungi	≤ 100 cfu/g	Complies
Salmonella	Negative	Complies
E. coli	Negative	Complies
Storage	Store in cool & dry place. Do not freeze.<br>Keep away from strong light and heat.	
Shelf life	2 years when properly stored.	

*No.*72 *Platycodon grandiflorus* extract

**Product Name**	*Platycodon grandiflorus* extract	
Lot Number	T0288	
Quantity	1500 kg	
Manufacture Date	2022-04-18	
Testing Date	2022-04-20	
**Analysis**	**Specification**	**Results**
Appearance	Brown-yellow powder	Complies
Ash	≤ 10.0%	3.53%
Moisture	≤ 7.0%	2.45%
Heavy metals	≤ 10 ppm	Complies
Odor	Characteristic	Complies
Particle size	100% through 80 mesh	Complies
**Microbiological**		
Total of bacteria	≤ 1000 cfu/g	Complies
Fungi	≤ 100 cfu/g	Complies
Salmonella	Negative	Complies
E. coli	Negative	Complies
Storage	Store in cool & dry place. Do not freeze. Keep away from strong light and heat.	
Shelf life	2 years when properly stored.	

*No.*73 *Cinnamomum cassia* extract

**Product Name**	*Cinnamomum cassia* extract	
Lot Number	T0329	
Quantity	1300 kg	
Manufacture Date	2022-04-25	
Testing Date	2022-04-27	
**Analysis**	**Specification**	**Results**
Appearance	Brown-red powder	Complies
Ash	≤ 10.0%	3.68%
Moisture	≤ 7.0%	2.46%
Heavy metals	≤ 10 ppm	Complies
Odor	Characteristic	Complies
Particle size	100% through 80 mesh	Complies
**Microbiological**		
Total of bacteria	≤ 1000 cfu/g	Complies
Fungi	≤ 100 cfu/g	Complies
Salmonella	Negative	Complies
E. coli	Negative	Complies
Storage	Store in cool & dry place. Do not freeze.<br>Keep away from strong light and heat.	
Shelf life	2 years when properly stored.	

*No.*74 *Paeonia lactiflora* extract

**Product Name**	*Paeonia lactiflora* extract	
Lot Number	T0295	
Quantity	500 kg	
Manufacture Date	2022-05-06	
Testing Date	2022-05-06	
**Analysis**	**Specification**	**Results**
Appearance	Brown powder	Complies
Ash	≤ 5.0%	4.23%
Moisture	≤ 5.0%	3.46%
Pesticides	Negative	Complies
Heavy metals	≤ 10 ppm	Complies
Pb	≤ 2.0 ppm	Complies
As	≤ 2.0 ppm	Complies
Odor	Characteristic	Complies
Particle size	100% through 80 mesh	Complies
**Microbiological**		
Total of bacteria	≤ 1000 cfu/g	Complies
Fungi	≤ 100 cfu/g	Complies
Salmonella	Negative	Complies
E. coli	Negative	Complies
Storage Store in cool & dry place. Do not freeze. Keep away from strong light and heat.		
Shelf life 2 years when properly stored.		

*No.*75 *Astragalus membranaceus* extract

**Product Name**	*Astragalus membranaceus* extract	
Lot Number	T0330	
Quantity	100 kg	
Manufacture Date	2022-04-19	
Testing Date	2022-04-21	
**Analysis**	**Specification**	**Results**
Appearance	Brown fine powder	Complies
Moisture	≤ 5.0%	3.5%
Heavy metals	≤ 20 ppm	Complies
Odor	Characteristic	Complies
Particle size	100% through 80 mesh	Complies
**Microbiological**		
Total of bacteria	≤ 1000 cfu/g	Complies
Fungi	≤ 100 cfu/g	Complies
Salmonella	Negative	Complies
E. coli	Negative	Complies
Storage	Store in cool & dry place. Do not freeze.<br>Keep away from strong light and heat.	
Shelf life	2 years when properly stored.	

*No.*76 *Scutellariae radix* extract

**Product Name**	*Scutellariae radix* extract	
Lot Number	T0331	
Quantity	600kg	
Manufacture Date	2022-04-19	
Testing Date	2022-04-21	
**Analysis**	**Specification**	**Results**
Appearance	Brown fine powder	Complies
Ash	≤ 10.0%	3.15%
Moisture	≤ 7.0%	3.73%
Heavy metals	≤ 10 ppm	Complies
Odor	Characteristic	Complies
Particle size	100% through 80 mesh	Complies
**Microbiological**		
Total of bacteria	≤ 1000 cfu/g	Complies
Fungi	≤ 100 cfu/g	Complies
Salmonella	Negative	Complies
E. coli	Negative	Complies
Storage	Store in cool & dry place. Do not freeze.<br>Keep away from strong light and heat.	
Shelf life	2 years when properly stored.	

*No.*77 ***Pueraria lobata*** **extract**

**Product Name**	*Pueraria lobata* extract	
Lot Number	T0081	
Quantity	100 kg	
Manufacture Date	2022-04-14	
Testing Date	2022-04-17	
**Analysis**	**Specification**	**Results**
Appearance	Brown fine powder	Complies
Ash	≤ 5.0%	3.65%
Moisture	≤ 5.0%	2.5%
Heavy metals	≤ 10 ppm	Complies
Odor	Characteristic	Complies
Particle size	100% through 80 mesh	Complies
**Microbiological**		
Total of bacteria	≤ 1000 cfu/g	Complies
Fungi	≤ 100 cfu/g	Complies
Salmonella	Negative	Complies
E. coli	Negative	Complies
Storage	Store in cool & dry place. Do not freeze.<br>Keep away from strong light and heat.	
Shelf life	2 years when properly stored.	

*No.*78 ***Lonicera japonica*** **extract**

**Product Name**	*Lonicera japonica* extract	
Lot Number	T0289	
Quantity	500 kg	
Manufacture Date	2022-06-08	
Testing Date	2022-06-09	
**Analysis**	**Specification**	**Results**
Appearance	Brown-yellow fine powder	Complies
Ash	≤ 5.0%	2.28%
Moisture	≤ 5.0%	3.35%
Pesticides	Negative	Complies
Heavy metals	≤ 10 ppm	Complies
Odor	Characteristic	Complies
Particle size	100% through 80 mesh	Complies
**Microbiological**		
Total of bacteria	≤ 1000 cfu/g	Complies
Fungi	≤ 100 cfu/g	Complies
Salmonella	Negative	Complies
E. coli	Negative	Complies
Storage	Store in cool & dry place. Do not freeze.<br>Keep away from strong light and heat.	
Shelf life	2 years when properly stored.	

No.79 Zingiber officinale extract

**Product Name**	*Zingiber officinale* extract	
Lot Number	T0296	
Quantity	1200 kg	
Manufacture Date	2022-02-21	
Testing Date	2022-02-23	
**Analysis**	**Specification**	**Results**
Appearance	Yellow-brown powder	Complies
Ash	≤ 7.0%	3.15%
Moisture	≤ 5.0%	2.23%
Heavy metals	≤ 20 ppm	Complies
Odor	Characteristic	Complies
Particle size	100% through 80 mesh	Complies
**Microbiological**		
Total of bacteria	≤ 1000 cfu/g	Complies
Fungi	≤ 100 cfu/g	Complies
Salmonella	Negative	Complies
E. coli	Negative	Complies
Storage	Store in cool & dry place. Do not freeze.<br>Keep away from strong light and heat.	
Shelf life	2 years when properly stored.	

*No.*80 *Sophora flavescens* extract

**Product Name**	*Sophora flavescens* extract	
Lot Number	T0224	
Quantity	540 kg	
Manufacture Date	2022-03-11	
Testing Date	2022-03-13	
**Analysis**	**Specification**	**Results**
Appearance	Brown fine powder	Complies
Ash	≤ 10.0%	3.95%
Moisture	≤ 7.0%	3.56%
Heavy metals	≤ 10 ppm	Complies
Odor	Characteristic	Complies
Particle size	100% through 80 mesh	Complies
**Microbiological**		
Total of bacteria	≤ 1000 cfu/g	Complies
Fungi	≤ 100 cfu/g	Complies
Salmonella	Negative	Complies
E. coli	Negative	Complies
Storage	Store in cool & dry place. Do not freeze.<br>Keep away from strong light and heat.	
Shelf life	2 years when properly stored.	

*No.*81 *Allium sativum* extract

**Product Name**	*Allium sativum* extract	
Lot Number	T0279	
Manufacture Date	2022-05-07	
Testing Date	2022-05-09	
**Analysis**	**Specification**	**Results**
Appearance	Off-white fine powder	Complies
Ash	≤ 5.0%	2.3%
Moisture	≤ 5.0%	4.0%
Pesticides	Negative	Complies
Heavy metals	≤ 10 ppm	Complies
Pb	≤ 2.0 ppm	Complies
As	≤ 2.0 ppm	Complies
Hg	≤ 0.5 ppm	Complies
Odor	Characteristic	Complies
Particle size	100% through 80 mesh	Complies
**Microbiological**		
Total of bacteria	≤ 1000 cfu/g	Complies
Fungi	≤ 100 cfu/g	Complies
Salmonella	Negative	Complies
E. coli	Negative	Complies
Storage	Store in cool & dry place. Do not freeze.<br>Keep away from strong light and heat.	
Shelf life	2 years when properly stored.	

*No.*82 *Rheum officinale* extract

**Product Name**	*Rheum officinale* extract	
Lot Number	T0280	
Manufacture Date	2022-02-19	
Testing Date	2022-02-22	
**Analysis**	**Specification**	**Results**
Appearance	Yellow to brown fine powder	Complies
Moisture	≤ 10.0%	3.58%
Heavy metals	≤ 10 ppm	Complies
As	≤ 2.0 ppm	Complies
Odor	Characteristic	Complies
Particle size	100% through 80 mesh	Complies
**Microbiological**		
Total of bacteria	≤ 1000 cfu/g	Complies
Fungi	≤ 100 cfu/g	Complies
Salmonella	Negative	Complies
E. coli	Negative	Complies
Storage	Store in cool & dry place. Do not freeze.<br>Keep away from strong light and heat.	
Shelf life	2 years when properly stored.	

*No.*83 *Cnidium monnieri* extract

**Product Name**	*Cnidium monnieri* extract	
DST220319	T0297	
2022-03-19	2022-03-19	
2022-03-21	2022-03-21	
**Analysis**	**Specification**	**Results**
Appearance	Brown fine powder	Complies
Ash	≤ 10.0%	3.48%
Moisture	≤ 7.0%	3.62%
Pesticides	≤ 2.0 ppm	Complies
Heavy metals	≤ 10 ppm	Complies
Odor	Characteristic	Complies
Particle size	100% through 80 mesh	Complies
**Microbiological**		
Total of bacteria	≤ 1000 cfu/g	Complies
Fungi	≤ 100 cfu/g	Complies
Salmonella	Negative	Complies
E. coli	Negative	Complies
Storage	Store in cool & dry place. Do not freeze.<br>Keep away from strong light and heat.	
Shelf life	2 years when properly stored.	

*No.*84 *Angelica sinensis* extract

**Product Name**	*Angelica sinensis* extract	
Lot Number	T0281	
Manufacture Date	2022-05-23	
Testing Date	2022-05-26	
**Analysis**	**Specification**	**Results**
Appearance	Yellow-brown powder	Complies
Ash	≤ 10.0%	4.80%
Moisture	≤ 7.0%	3.82%
Heavy metals	≤ 10 ppm	Complies
Odor	Characteristic	Complies
Particle size	100% through 80 mesh	Complies
**Microbiological**		
Total of bacteria	≤ 1000 cfu/g	Complies
Fungi	≤ 100 cfu/g	Complies
Salmonella	Negative	Complies
E. coli	Negative	Complies
Storage	Store in cool & dry place. Do not freeze.<br>Keep away from strong light and heat.	
Shelf life	2 years when properly stored.	

*No.*85 *Tribulus terrestris* extract

**Product Name**	*Tribulus terrestris* extract	
Lot Number	T0347	
Manufacture Date	2021.08.04	
**Analysis**	**Specification**	**Results**
Appearance	Brown powder	Complies
Moisture	≤ 5.0%	3.81%
Ash	≤ 5.0%	2.10%
Heavy metals	≤ 10 ppm	Complies
Odor	Unique smell	Complies
Taste	Unique taste	Complies
Particle size	95% through 80 mesh	Complies
**Microbiological**		
Total of bacteria	≤ 1000 cfu/g	Complies
Yeast	≤ 100 cfu/g	Complies
Fungi	≤ 100 cfu/g	Complies
Salmonella	Negative	Complies
E. coli	< 3.0 MPN/g	Complies
Storage	Store in cool & dry place. Do not freeze.<br>Keep away from strong light and heat.	
Shelf life	2 years when properly stored.	

*No.*86 *Glycyrrhiza uralensis* extract

**Product Name**	*Glycyrrhiza uralensis* extract	
Lot Number	T0064	
Quantity	800 kg	
Manufacture Date	2022-02-15	
Testing Date	2022-02-19	
**Analysis**	**Specification**	**Results**
Appearance	Yellow brown powder	Complies
Ash	≤ 8.0%	3.88%
Loss on Drying	≤ 5.0%	3.25%
Heavy metals	≤ 10 ppm	Complies
Pb	≤ 2.0 ppm	Complies
As	≤ 2.0 ppm	Complies
Odor	Characteristic	Complies
Particle size	100% through 80 mesh	Complies
Bulk density	40 - 60 g/100 mL	50 g/100 mL
**Microbiological**		
Total of bacteria	≤ 1000 cfu/g	Complies
Fungi	≤ 100 cfu/g	Complies
Salmonella	Negative	Complies
E. coli	Negative	Complies
Staph. aureus	Negative	Complies
Storage Store in cool & dry place. Do not freeze. Keep away from strong light and heat. Shelf life 2 years when properly stored.		

*No.*87 *Magnolia officinalis* extract

**Product Name**	*Magnolia officinalis* extract	
Lot Number	T0332	
Quantity	800 kg	
Manufacture Date	2022-03-19	
Testing Date	2022-03-21	
**Analysis**	**Specification**	**Results**
Appearance	Brown powder	Complies
Ash	≤ 5.0 %	2.46%
Moisture	≤ 5.0%	2.31%
Heavy metals	≤ 10 ppm	Complies
Odor	Unique smell	Complies
Taste	Unique taste	Complies
Particle size	95% through 80 mesh	Complies
**Microbiological**		
Total of bacteria	≤ 1000 cfu/g	Complies
Fungi	≤ 100 cfu/g	Complies
Yeast	≤ 100 cfu/g	Complies
Salmonella	Negative	Complies
E. coli	Negative	Complies
Staph. aureus	Negative	Complies
Storage Store in cool & dry place. Do not freeze.<br>Keep away from strong light and heat.<br>Shelf life 2 years when properly stored.		

*No.*88 *Schisandra chinensis* extract

**Product Name**	*Schisandra chinensis* extract	
Lot Number	T0326	
Quantity	350 kg	
Manufacture Date	2022-02-18	
Testing Date	2022-02-19	
**Analysis**	**Specification**	**Results**
Appearance	Light yellow or yellow brown fine powder	Complies
Moisture	≤ 5.0%	3.65%
Pesticides	Negative	Complies
Heavy metals	≤ 10 ppm	Complies
Odor	Characteristic	Complies
Particle size	100% through 80 mesh	Complies
**Microbiological**		
Total of bacteria	≤ 1000 cfu/g	Complies
Fungi	≤ 100 cfu/g	Complies
Salmonella	Negative	Complies
E. coli	Negative	Complies
Storage	Store in cool & dry place. Do not freeze.<br>Keep away from strong light and heat.	
Shelf life	2 years when properly stored.	

*No.*89 *Lycium ruthenicum* extract

**Product Name**	*Lycium ruthenicum* extract	
Lot Number	T0333	
Quantity	220 kg	
Manufacture Date	2022-04-25	
Testing Date	2022-04-26	
**Analysis**	**Specification**	**Results**
Appearance	Deep black fine powder	Complies
Loss on Drying	≤ 5.0%	4.78%
Ash	≤ 5.0%	3.45%
Bulk Density	40 - 60 g/100 mL	45 g/100 mL
Heavy metals	≤ 10 ppm	Complies
Pb	≤ 2 ppm	Complies
As	≤ 2 ppm	Complies
Hg	≤ 1 ppm	Complies
Odor	Characteristic	Complies
Particle size	100% through 80 mesh	Complies
**Microbiological**		
Total of bacteria	≤ 5000 cfu/g	Complies
Total Yeast & Mold	≤ 300 cfu/g	Complies
Salmonella	Negative	Complies
E. coli	Negative	Complies
Staph. aureus	Negative	Complies
Storage	Store in cool & dry place. Do not freeze.<br>Keep away from strong light and heat.	
Shelf life	2 years when properly stored.	

*No.*90 *Gynostemma pentaphyllum* extract

**Product Name**	*Gynostemma pentaphyllum* extract	
Lot Number	T0290	
Quantity	660 kg	
Manufacture Date	2022-05-22	
Testing Date	2022-05-24	
**Analysis**	**Specification**	**Results**
Appearance	Light yellow powder	Complies
Ash	≤ 10.0%	4.35%
Moisture	≤ 7.0%	4.31%
Pesticides	Negative	Complies
Heavy metals	≤ 10 ppm	Complies
Odor	Characteristic	Complies
Particle size	100% through 80 mesh	Complies
**Microbiological**		
Total of bacteria	≤ 1000 cfu/g	Complies
Fungi	≤ 100 cfu/g	Complies
Salmonella	Negative	Complies
E. coli	Negative	Complies
Storage	Store in cool & dry place. Do not freeze.<br>Keep away from strong light and heat.	
Shelf life	2 years when properly stored.	

*No.*91 *Hypericum perforatum* extract

**Product Name**	*Hypericum perforatum* extract	
Lot Number	T0286	
Manufacture Date	2022-06-03	
Testing Date	2022-06-04	
**Analysis**	**Specification**	**Results**
Appearance	Brown-black fine powder	Complies
Ash	≤ 5.0%	3.06%
Moisture	≤ 5.0%	3.15%
Pesticides	Negative	Complies
Heavy metals	≤ 10 ppm	Complies
Odor	Characteristic	Complies
Particle size	100% through 80 mesh	Complies
**Microbiological**		
Total of bacteria	≤ 1000 cfu/g	Complies
Fungi	≤ 100 cfu/g	Complies
Salmonella	Negative	Complies
E. coli	Negative	Complies
Storage	Store in cool & dry place. Do not freeze. Keep away from strong light and heat.	
Shelf life	2 years when properly stored.	

*No.*92 *Citrus aurantium* extract

**Product Name**	*Citrus aurantium* extract	
Lot Number	T0309	
Manufacture Date	2022-04-11	
Testing Date	2022-04-15	
**Analysis**	**Specification**	**Results**
Appearance	Light brown fine powder	Complies
Ash	≤ 10.0%	2.98%
Moisture	≤ 7.0%	4.67%
Heavy metals	≤ 10 ppm	Complies
Odor	Characteristic	Complies
Particle size	100% through 80 mesh	Complies
**Microbiological**		
Total of bacteria	≤ 1000 cfu/g	Complies
Fungi	≤ 100 cfu/g	Complies
Salmonella	Negative	Complies
E. coli	Negative	Complies
Storage	Store in cool & dry place. Do not freeze. Keep away from strong light and heat.	
Shelf life	2 years when properly stored.	

*No.*93 *Equisetum arvense* extract

**Product Name**	*Equisetum arvense* extract	
Lot Number	T0291	
Quantity	980 kg	
Manufacture Date	2022-04-21	
Testing Date	2022-04-23	
**Analysis**	**Specification**	**Results**
Appearance	Yellow-brown fine powder	Complies
Loss on Drying	≤ 5.0%	2.88%
Ash	≤ 5.0%	3.26%
Heavy metals	≤ 10 ppm	Complies
Pb	≤ 2 ppm	Complies
Cd	≤ 0.2 ppm	Complies
Hg	≤ 0.1 ppm	Complies
Odor	Characteristic	Complies
Particle size	100% through 80 mesh	Complies
**Microbiological**		
Total of bacteria	≤ 1000 cfu/g	Complies
Fungi	≤ 100 cfu/g	Complies
Salmonella	Negative	Complies
E. coli	Negative	Complies
Staph. aureus	Negative	Complies
Storage	Store in cool & dry place. Do not freeze.<br>Keep away from strong light and heat.	
Shelf life	2 years when properly stored.	

*No.*94 *Gardenia jasminoides* extract

**Product Name**	*Gardenia jasminoides* extract	
Lot Number	T0311	
Quantity	800 kg	
Manufacture Date	2022-05-08	
Testing Date	2022-05-13	
**Analysis**	**Specification**	**Results**
Appearance	Brown-yellow fine powder	Complies
Ash	≤ 10.0%	4.25%
Moisture	≤ 7.0%	3.19%
Heavy metals	≤ 10 ppm	Complies
Odor	Characteristic	Complies
Particle size	100% through 80 mesh	Complies
**Microbiological**		
Total of bacteria	≤ 1000 cfu/g	Complies
Fungi	≤ 100 cfu/g	Complies
Salmonella	Negative	Complies
E. coli	Negative	Complies
Storage	Store in cool & dry place. Do not freeze.<br>Keep away from strong light and heat.	
Shelf life	2 years when properly stored.	

*No.*95 *Punica granatum* extract

**Product Name**	*Punica granatum* extract	
Lot Number	T0298	
Quantity	900 kg	
Manufacture Date	2022-06-11	
Testing Date	2022-06-15	
**Analysis**	**Specification**	**Results**
Appearance	Brown-yellow fine powder	Complies
Ash	≤ 10.0%	4.85%
Moisture	≤ 7.0%	3.66%
Heavy metals	≤ 10 ppm	Complies
Odor	Characteristic	Complies
Particle size	100% through 80 mesh	Complies
**Microbiological**		
Total of bacteria	≤ 1000 cfu/g	Complies
Fungi	≤ 100 cfu/g	Complies
Salmonella	Negative	Complies
E. coli	Negative	Complies
Storage	Store in cool & dry place. Do not freeze.<br>Keep away from strong light and heat.	
Shelf life	2 years when properly stored.	

*No.*96 *Trifolium pratense* extract

**Product Name**	*Trifolium pratense* extract	
Lot Number	T0334	
Quantity	700 kg	
Manufacture Date	2022-05-09	
Testing Date	2022-05-11	
**Analysis**	**Specification**	**Results**
Appearance	Brown fine powder	Complies
Ash	≤ 10.0%	4.25%
Moisture	≤ 7.0%	4.37%
Heavy metals	≤ 10 ppm	Complies
Odor	Characteristic	Complies
Particle size	100% through 80 mesh	Complies
**Microbiological**		
Total of bacteria	≤ 1000 cfu/g	Complies
Fungi	≤ 100 cfu/g	Complies
Salmonella	Negative	Complies
E. coli	Negative	Complies
Storage	Store in cool & dry place. Do not freeze.<br>Keep away from strong light and heat.	
Shelf life	2 years when properly stored.	

*No.*97 *Camellia sinensis* extract

**Product Name**	*Camellia sinensis* extract	
Lot Number	T0351	
Quantity	900 kg	
Manufacture Date	2022-04-21	
Testing Date	2022-04-23	
**Analysis**	**Specification**	**Results**
Appearance	Brown-yellow powder	Complies
Loss on Drying	≤ 5%	3.31%
Ash	≤ 5%	2.35%
BHC	≤ 0.2 ppm	Complies
DDT	≤ 0.2 ppm	Complies
Heavy metals	≤ 10 ppm	Complies
Residual Solvents	≤ 0.05%	Complies
Particle size	100% through 80 mesh	Complies
**Microbiological**		
Total of bacteria	≤ 1000 cfu/g	Complies
Fungi	≤ 25 cfu/g	Complies
Yeast	≤ 25 cfu/g	Complies
Salmonella	Negative	Complies
E. coli	Negative	Complies
Storage	Store in cool & dry place. Do not freeze. Keep away from strong light and heat.	
Shelf life	2 years when properly stored.	

*No.*98 *Phaseolus vulgaris* extract

**Product Name**	*Phaseolus vulgaris* extract	
Lot Number	DB0358	
Manufacture Date	2022-05-16	
Testing Date	2022-05-18	
**Analysis**	**Specification**	**Results**
Appearance	White fine powder	Complies
Ash	≤ 10.0%	3.95%
Moisture	≤ 7.0%	4.16%
Heavy metals	≤ 10 ppm	Complies
Odor	Characteristic	Complies
Particle size	100% through 80 mesh	Complies
**Microbiological**		
Total of bacteria	≤ 1000 cfu/g	Complies
Fungi	≤ 100 cfu/g	Complies
Salmonella	Negative	Complies
E. coli	Negative	Complies
Storage	Store in cool & dry place. Do not freeze.<br>Keep away from strong light and heat.	
Shelf life	2 years when properly stored.	

*No.*99 *Brassica oleracea* extract

**Product Name**	*Brassica oleracea* extract	
Lot Number	T0318	
Manufacture Date	2022-03-19	
Testing Date	2022-03-21	
**Analysis**	**Specification**	**Results**
Appearance	Brown fine powder	Complies
Ash	≤ 10.0%	3.25%
Moisture	≤ 7.0%	2.78%
Pesticides	Negative	Complies
Heavy metals	≤ 10 ppm	Complies
Odor	Characteristic	Complies
Particle size	100% through 80 mesh	Complies
**Microbiological**		
Total of bacteria	≤ 10000 cfu/g	Complies
Fungi	≤ 1000 cfu/g	Complies
Salmonella	Negative	Complies
E. coli	Negative	Complies
Storage	Store in cool & dry place. Do not freeze.<br>Keep away from strong light and heat.	
Shelf life	2 years when properly stored.	

*No.*100 *Prunella vulgaris* extract

**Product Name**	*Prunella vulgaris* extract	
Lot Number	T0343	
Quantity	1500 kg	
Manufacture Date	2022-02-16	
Testing Date	2022-02-18	
**Analysis**	**Specification**	**Results**
Appearance	Brown-yellow powder	Complies
Ash	≤ 10.0%	4.95%
Moisture	≤ 7.0%	3.78%
Heavy metals	≤ 10 ppm	Complies
Odor	Characteristic	Complies
Particle size	100% through 80 mesh	Complies
**Microbiological**		
Total of bacteria	≤ 10000 cfu/g	Complies
Fungi	≤ 1000 cfu/g	Complies
Salmonella	Negative	Complies
E. coli	Negative	Complies
Storage	Store in cool & dry place. Do not freeze.<br>Keep away from strong light and heat.	
Shelf life	2 years when properly stored.	

*No.*101 *Ziziphi spinosae* extract

**Product Name**	*Ziziphi spinosae* extract	
Lot Number	T0299	
Quantity	600 kg	
Manufacture Date	2022-05-17	
Testing Date	2022-05-11	
**Analysis**	**Specification**	**Results**
Appearance	Brown fine powder	Complies
Ash	≤ 10.0%	3.95%
Moisture	≤ 7.0%	3.27%
Heavy metals	≤ 10 ppm	Complies
Odor	Characteristic	Complies
Particle size	100% through 80 mesh	Complies
**Microbiological**		
Total of bacteria	≤ 10000 cfu/g	Complies
Fungi	≤ 1000 cfu/g	Complies
Salmonella	Negative	Complies
E. coli	Negative	Complies
Storage	Store in cool & dry place. Do not freeze.<br>Keep away from strong light and heat.	
Shelf life	2 years when properly stored.	

*No.*102 *Cannabis fructus* extract

**Product Name**	*Cannabis fructus* extract	
Lot Number	T0335	
Quantity	600 kg	
Manufacture Date	2022-04-16	
Testing Date	2022-04-16	
**Analysis**	**Specification**	**Results**
Appearance	Brown powder	Complies
Ash	≤ 10.0%	4.25%
Moisture	≤ 7.0%	2.85%
Heavy metals	≤ 10 ppm	Complies
Odor	Characteristic	Complies
Particle size	100% through 80 mesh	Complies
**Microbiological**		
Total of bacteria	≤ 10000 cfu/g	Complies
Fungi	≤ 1000 cfu/g	Complies
Salmonella	Negative	Complies
E. coli	Negative	Complies
Storage Store in cool & dry place. Do not freeze.<br>Keep away from strong light and heat.<br>Shelf life 2 years when properly stored.		

*No.*103 *Fructus mori* extract

**Product Name**	*Fructus mori* extract	
Lot Number	T0300	
Quantity	1500 kg	
Manufacture Date	2022-04-19	
Testing Date	2022-04-21	
**Analysis**	**Specification**	**Results**
Appearance	Purple-red powder	Complies
Ash	≤ 10.0%	3.57%
Moisture	≤ 7.0%	4.23%
Heavy metals	≤ 10 ppm	Complies
Odor	Characteristic	Complies
Particle size	100% through 80 mesh	Complies
**Microbiological**		
Total of bacteria	≤ 10000 cfu/g	Complies
Fungi	≤ 1000 cfu/g	Complies
Salmonella	Negative	Complies
E. coli	Negative	Complies
Storage	Store in cool & dry place. Do not freeze. Keep away from strong light and heat.	
Shelf life	2 years when properly stored.	

*No.*104 *Ramulus mori* extract

**Product Name**	*Ramulus mori* extract	
Lot Number	T0301	
Quantity	790 kg	
Manufacture Date	2022-04-19	
Testing Date	2022-04-21	
**Analysis**	**Specification**	**Results**
Appearance	Brown powder	Complies
Ash	≤ 10.0%	4.88%
Moisture	≤ 7.0%	2.74%
Heavy metals	≤ 10 ppm	Complies
Odor	Characteristic	Complies
Particle size	100% through 80 mesh	Complies
**Microbiological**		
Total of bacteria	≤ 10000 cfu/g	Complies
Fungi	≤ 1000 cfu/g	Complies
Salmonella	Negative	Complies
E. coli	Negative	Complies
Storage	Store in cool & dry place. Do not freeze.<br>Keep away from strong light and heat.	
Shelf life	2 years when properly stored.	

*No.*105 *Rhamnus utilis* extract

**Product Name**	*Rhamnus utilis* extract	
Lot Number	T0302	
Quantity	680 kg	
Manufacture Date	2022-05-06	
Testing Date	2022-05-09	
**Analysis**	**Specification**	**Results**
Appearance	Brown-yellow powder	Complies
Ash	≤ 10.0%	4.19%
Moisture	≤ 7.0%	3.69%
Heavy metals	≤ 10 ppm	Complies
Odor	Characteristic	Complies
Particle size	100% through 80 mesh	Complies
**Microbiological**		
Total of bacteria	≤ 10000 cfu/g	Complies
Fungi	≤ 1000 cfu/g	Complies
Salmonella	Negative	Complies
E. coli	Negative	Complies
Storage Store in cool & dry place. Do not freeze.<br>Keep away from strong light and heat.<br>Shelf life 2 years when properly stored.		

*No.*106 *Passiflora caerulea* extract

**Product Name**	*Passiflora caerulea* extract	
Lot Number	T0344	
Quantity	980 kg	
Manufacture Date	2022-05-16	
Testing Date	2022-05-18	
**Analysis**	**Specification**	**Results**
Appearance	Brown powder	Complies
Ash	≤ 10.0%	3.98%
Moisture	≤ 7.0%	4.71%
Heavy metals	≤ 10 ppm	Complies
Odor	Characteristic	Complies
Particle size	100% through 80 mesh	Complies
**Microbiological**		
Total of bacteria	≤ 10000 cfu/g	Complies
Fungi	≤ 1000 cfu/g	Complies
Salmonella	Negative	Complies
E. coli	Negative	Complies
Storage Store in cool & dry place. Do not freeze.<br>Keep away from strong light and heat.<br>Shelf life 2 years when properly stored.		

*No.*107 *Ophiopogon japonicus* extract

**Product Name**	*Ophiopogon japonicus* extract	
Lot Number	T0272	
Quantity	800 kg	
Manufacture Date	2022-04-11	
Testing Date	2022-04-12	
**Analysis**	**Specification**	**Results**
Appearance	Brown-yellow powder	Complies
Ash	≤ 10.0%	3.66%
Moisture	≤ 7.0%	3.61%
Heavy metals	≤ 10 ppm	Complies
Odor	Characteristic	Complies
Particle size	100% through 80 mesh	Complies
**Microbiological**		
Total of bacteria	≤ 10000 cfu/g	Complies
Fungi	≤ 1000 cfu/g	Complies
Salmonella	Negative	Complies
E. coli	Negative	Complies
Storage	Store in cool & dry place. Do not freeze. Keep away from strong light and heat.	
Shelf life	2 years when properly stored.	

*No.*108 *Coix lacryma-jobi L. var. ma-yuen* extract

**Product Name**	*Coix lacryma-jobi L. var. ma-yuen* extract	
Lot Number	T0319	
Manufacture Date	2022-02-22	
Testing Date	2022-02-25	
**Analysis**	**Specification**	**Results**
Appearance	Brown powder	Complies
Ash	≤ 10.0%	3.95%
Moisture	≤ 7.0%	3.87%
Heavy metals	≤ 10 ppm	Complies
Odor	Characteristic	Complies
Particle size	100% through 80 mesh	Complies
**Microbiological**		
Total of bacteria	≤ 10000 cfu/g	Complies
Fungi	≤ 1000 cfu/g	Complies
Salmonella	Negative	Complies
E. coli	Negative	Complies
Storage Store in cool & dry place. Do not freeze.<br>Keep away from strong light and heat.<br>Shelf life 2 years when properly stored.		

*No.*109 *Rehmannia glutinosa* extract

**Product Name**	*Rehmannia glutinosa* extract	
Lot Number	T0303	
Quantity	530 kg	
Manufacture Date	2022-03-28	
Testing Date	2022-04-06	
**Analysis**	**Specification**	**Results**
Appearance	Yellow brown powder	Complies
Ash	≤ 8.0%	4.68%
Moisture	≤ 5.0%	3.89%
Heavy metals	≤ 10 ppm	Complies
Pb	≤ 2.0 ppm	Complies
As	≤ 2.0 ppm	Complies
Odor	Characteristic	Complies
Particle size	100% through 80 mesh	Complies
**Microbiological**		
Total of bacteria	≤ 1000 cfu/g	Complies
Fungi	≤ 100 cfu/g	Complies
Salmonella	Negative	Complies
E. coli	Negative	Complies
Storage Store in cool & dry place. Do not freeze.<br>Keep away from strong light and heat.<br>Shelf life 2 years when properly stored.		

*No.*110 *Ziziphus jujuba* extract

**Product Name**	*Ziziphus jujuba* extract	
Lot Number	T0282	
Quantity	1160 kg	
Manufacture Date	2022-04-24	
Testing Date	2022-04-26	
**Analysis**	**Specification**	**Results**
Appearance	Light brown powder	Complies
Loss on Drying	≤ 5.0%	3.71%
Ash	≤ 5.0%	2.18%
Heavy metals	≤ 10 ppm	Complies
Pb	≤ 2 ppm	Complies
As	≤ 2 ppm	Complies
Cd	≤ 0.2 ppm	Complies
Hg	≤ 0.1 ppm	Complies
Odor	Characteristic	Complies
Particle size	98% through 80 mesh	Complies
**Microbiological**		
Total of bacteria	≤ 1000 cfu/g	Complies
Fungi	≤ 100 cfu/g	Complies
Salmonella	Negative	Complies
E. coli	Negative	Complies
Staph. aureus	Negative	Complies
Storage	Store in cool & dry place. Do not freeze.<br>Keep away from strong light and heat.	
Shelf life	2 years when properly stored.	

*No.*111 *Phellodendron chinense* extract

**Product Name**	*Phellodendron chinense* extract	
Lot Number	T0336	
Quantity	980 kg	
Manufacture Date	2021-08-06	
Testing Date	2021-08-12	
**Analysis**	**Specification**	**Results**
Appearance	Yellow to yellowish brown powder	Complies
Ash	≤ 5.0%	4.23%
Moisture	≤ 5.0%	3.46%
Heavy metals	≤ 10 ppm	Complies
Pb	≤ 2 ppm	Complies
As	≤ 2 ppm	Complies
Odor	Characteristic	Complies
Particle size	100% through 80 mesh	Complies
**Microbiological**		
Total of bacteria	≤ 1000 cfu/g	Complies
Fungi	≤ 100 cfu/g	Complies
Salmonella	Negative	Complies
E. coli	Negative	Complies
Storage Store in cool & dry place. Do not freeze.<br>Keep away from strong light and heat.<br>Shelf life 2 years when properly stored.		

*No.*112 *Dichroae radix* extract

**Product Name**	*Dichroae radix* extract	
Lot Number	T0348	
Quantity	2000 kg	
Manufacture Date	2022-06-18	
Testing Date	2022-06-26	
**Analysis**	**Specification**	**Results**
Appearance	Brown fine powder	Complies
Moisture	≤ 7.0%	3.45%
Heavy metals	≤ 10 ppm	Complies
Odor	Characteristic	Complies
Particle size	100% through 80 mesh	Complies
**Microbiological**		
Total of bacteria	≤ 10000 cfu/g	Complies
Fungi	≤ 1000 cfu/g	Complies
Salmonella	Negative	Complies
E. coli	Negative	Complies
Storage	Store in cool & dry place. Do not freeze.<br>Keep away from strong light and heat.	
Shelf life	2 years when properly stored.	

*No.*113 *Atractylodes macrocephala* extract

**Product Name**	*Atractylodes macrocephala* extract	
Lot Number	T0352	
Manufacture Date	2022-05-24	
Testing Date	2022-05-30	
**Analysis**	**Specification**	**Results**
Appearance	Yellow-brown powder	Complies
Ash	≤ 5.0%	3.21%
Moisture	≤ 5.0%	3.5%
Heavy metals	≤ 10 ppm	Complies
Odor	Characteristic	Complies
Particle size	100% through 80 mesh	Complies
**Microbiological**		
Total of bacteria	≤ 1000 cfu/g	Complies
Fungi	≤ 100 cfu/g	Complies
Salmonella	Negative	Complies
E. coli	Negative	Complies
Storage Store in cool & dry place. Do not freeze. Keep away from strong light and heat. Shelf life 2 years when properly stored.		

*No.*114 *Bletilla striata* extract

**Product Name**	*Bletilla striata* extract	
Lot Number	T0353	
Quantity	200 kg	
Manufacture Date	2022-05-21	
Testing Date	2022-05-21	
**Analysis**	**Specification**	**Results**
Appearance	Brown fine powder	Complies
Ash	≤ 5.0%	3.6%
Moisture	≤ 7.0%	3.8%
Heavy metals	≤ 10 ppm	Complies
Odor	Characteristic	Complies
Particle size	100% through 80 mesh	Complies
**Microbiological**		
Total of bacteria	≤ 10000 cfu/g	Complies
Fungi	≤ 1000 cfu/g	Complies
Salmonella	Negative	Complies
E. coli	Negative	Complies
Storage	Store in cool & dry place. Do not freeze. Keep away from strong light and heat.	
Shelf life	2 years when properly stored.	

*No.*115 *Agastache rugosa* extract

**Product Name**	*Agastache rugosa* extract	
Lot Number	T0337	
Quantity	570 kg	
Manufacture Date	2022-03-25	
Testing Date	2022-03-29	
**Analysis**	**Specification**	**Results**
Appearance	Brown powder	Complies
Ash	≤ 5.0%	3.4%
Moisture	≤ 5.0%	3.6%
Heavy metals	≤ 10 ppm	Complies
Pb	≤ 2.0 ppm	Complies
As	≤ 2.0 ppm	Complies
Hg	≤ 1.0 ppm	Complies
Odor	Characteristic	Complies
Particle size	100% through 80 mesh	Complies
**Microbiological**		
Total of bacteria	≤ 1000 cfu/g	Complies
Fungi	≤ 100 cfu/g	Complies
Salmonella	Negative	Complies
E. coli	Negative	Complies
Storage	Store in cool & dry place. Do not freeze. Keep away from strong light and heat.	
Shelf life	2 years when properly stored.	

*No.*116 *Xanthium strumarium* extract

**Product Name**	*Xanthium strumarium* extract	
Lot Number	T0349	
Quantity	400 kg	
Manufacture Date	2022-05-03	
Testing Date	2022-05-04	
**Analysis**	**Specification**	**Results**
Appearance	Brown powder	Complies
Ash	≤ 10.0%	3.1%
Moisture	≤ 7.0%	3.4%
Heavy metals	≤ 10 ppm	Complies
Pb	≤ 2.0 ppm	Complies
As	≤ 2.0 ppm	Complies
Hg	≤ 1.0 ppm	Complies
Odor	Characteristic	Complies
Particle size	100% through 80 mesh	Complies
**Microbiological**		
Total of bacteria	≤ 10000 cfu/g	Complies
Fungi	≤ 1000 cfu/g	Complies
Salmonella	Negative	Complies
E. coli	Negative	Complies
Storage Store in cool & dry place. Do not freeze.<br>Keep away from strong light and heat.<br>Shelf life 2 years when properly stored.		

*No.*117 *Citrus reticulata* extract

**Product Name**	*Citrus reticulata* extract	
Lot Number	T0350	
Quantity	1000 kg	
Manufacture Date	2021-12-28	
Testing Date	2022-01-06	
**Analysis**	**Specification**	**Results**
Appearance	Yellow-brown powder	Complies
Ash	≤ 5.0%	3.05%
Moisture	≤ 5.0%	3.55%
Heavy metals	≤ 10 ppm	Complies
Pb	≤ 2.0 ppm	Complies
As	≤ 2.0 ppm	Complies
Odor	Characteristic	Complies
Particle size	100% through 80 mesh	Complies
**Microbiological**		
Total of bacteria	≤ 1000 cfu/g	Complies
Fungi	≤ 100 cfu/g	Complies
Salmonella	Negative	Complies
E. coli	Negative	Complies
Storage Store in cool & dry place. Do not freeze.<br>Keep away from strong light and heat.<br>Shelf life 2 years when properly stored.		

*No.*118 *Coptis chinensis* extract

**Product Name**	*Coptis chinensis* extract	
Lot Number	T0354	
Quantity	500 kg	
Manufacture Date	2022-03-06	
Testing Date	2022-03-06	
**Analysis**	**Specification**	**Results**
Appearance	Brown powder	Complies
Ash	≤ 5.0%	4.23%
Moisture	≤ 5.0%	3.46%
Heavy metals	≤ 10 ppm	Complies
Pb	≤ 2.0 ppm	Complies
As	≤ 2.0 ppm	Complies
Odor	Characteristic	Complies
Particle size	100% through 80 mesh	Complies
**Microbiological**		
Total of bacteria	≤ 1000 cfu/g	Complies
Fungi	≤ 100 cfu/g	Complies
Salmonella	Negative	Complies
E. coli	Negative	Complies
Storage	Store in cool & dry place. Do not freeze. Keep away from strong light and heat.	
Shelf life	2 years when properly stored.	

*No.*119 *Apium graveolens* extract

**Product Name**	*Apium graveolens* extract	
Lot Number	T0355	
Manufacture Date	2021-06-26	
Testing Date	2021-06-30	
**Analysis**	**Specification**	**Results**
Appearance	Yellow-brown fine powder	Complies
Ash	≤ 5.0%	2.01%
Moisture	≤ 5.0%	3.75%
Heavy metals	≤ 10 ppm	Complies
Pb	≤ 3.0 ppm	Complies
As	≤ 1.0 ppm	Complies
Hg	≤ 0.2 ppm	Complies
Odor	Characteristic	Complies
Particle size	100% through 80 mesh	Complies
**Microbiological**		
Total of bacteria	≤ 3000 cfu/g	Complies
Fungi	≤ 100 cfu/g	Complies
Salmonella	Negative	Complies
E. coli	Negative	Complies
Storage	Store in cool & dry place. Do not freeze. Keep away from strong light and heat.	
Shelf life	2 years when properly stored.	

*No.*120 *Callerya reticulata* extract

**Product Name**	*Callerya reticulata* extract	
Lot Number	T0292	
Quantity	720 kg	
Manufacture Date	2022-03-17	
Testing Date	2022-03-23	
**Analysis**	**Specification**	**Results**
Appearance	Yellow-brown fine powder	Complies
Moisture	≤ 5.0%	3.6%
Ash	≤ 5.0%	3.0%
Heavy metals	≤ 10 ppm	Complies
Odor	Characteristic	Complies
Particle size	100% through 80 mesh	Complies
**Microbiological**		
Total of bacteria	≤ 3000 cfu/g	Complies
Fungi	≤ 100 cfu/g	Complies
Salmonella	Negative	Complies
E. coli	Negative	Complies
Storage	Store in cool & dry place. Do not freeze.<br>Keep away from strong light and heat.	
Shelf life	2 years when properly stored.	

*No.*121 *Panax notoginseng* extract

**Product Name**	*Panax notoginseng* extract	
Lot Number	T0304	
Quantity	780 kg	
Manufacture Date	2022-05-29	
Testing Date	2022-06-05	
**Analysis**	**Specification**	**Results**
Appearance	Yellow-brown fine powder	Complies
Moisture	≤ 5.0%	3.6%
Ash	≤ 5.0%	3.0%
Heavy metals	≤ 10 ppm	Complies
Odor	Characteristic	Complies
Particle size	100% through 80 mesh	Complies
**Microbiological**		
Total of bacteria	≤ 3000 cfu/g	Complies
Fungi	≤ 100 cfu/g	Complies
Salmonella	Negative	Complies
E. coli	Negative	Complies
Storage	Store in cool & dry place. Do not freeze.<br>Keep away from strong light and heat.	
Shelf life	2 years when properly stored.	

*No.*122 *Cornus officinalis* extract

**Product Name**	*Cornus officinalis* extract	
Lot Number	T0305	
Manufacture Date	2022-04-08	
Testing Date	2022-04-16	
**Analysis**	**Specification**	**Results**
Appearance	Yellow-brown powder	Complies
Ash	≤ 8.0%	4.08%
Moisture	≤ 5.0%	3.64%
Heavy metals	≤ 10 ppm	Complies
Pb	≤ 2.0 ppm	Complies
As	≤ 2.0 ppm	Complies
Odor	Characteristic	Complies
Particle size	100% through 80 mesh	Complies
**Microbiological**		
Total of bacteria	≤ 1000 cfu/g	Complies
Fungi	≤ 100 cfu/g	Complies
Salmonella	Negative	Complies
E. coli	Negative	Complies
Storage	Store in cool & dry place. Do not freeze.<br>Keep away from strong light and heat.	
Shelf life	2 years when properly stored.	

*No.*123 *Saposhnikovia divaricata* extract

**Product Name**	*Saposhnikovia divaricata* extract	
Lot Number	T0275	
Quantity	500 kg	
Manufacture Date	2022-01-12	
Testing Date	2022-01-19	
**Analysis**	**Specification**	**Results**
Appearance	Brown powder	Complies
Ash	≤ 5.0%	2.8%
Moisture	≤ 8.0%	3.6%
Heavy metals	≤ 20 ppm	Complies
Odor	Characteristic	Complies
Particle size	100% through 80 mesh	Complies
**Microbiological**		
Total of bacteria	≤ 1000 cfu/g	Complies
Fungi	≤ 100 cfu/g	Complies
Salmonella	Negative	Complies
E. coli	Negative	Complies
Storage	Store in cool & dry place. Do not freeze.<br>Keep away from strong light and heat.	
Shelf life	2 years when properly stored.	

*No.*124 *Perilla frutescens* extract

**Product Name**	*Perilla frutescens* extract	
Lot Number	T0313	
Quantity	1500 kg	
Manufacture Date	2022-03-13	
Testing Date	2022-03-19	
**Analysis**	**Specification**	**Results**
Appearance	Yellow-brown powder	Complies
Ash	≤ 5.0%	3.1%
Moisture	≤ 5.0%	3.2%
Heavy metals	≤ 10 ppm	Complies
Odor	Characteristic	Complies
Particle size	100% through 80 mesh	Complies
**Microbiological**		
Total of bacteria	≤ 3000 cfu/g	Complies
Fungi	≤ 100 cfu/g	Complies
Salmonella	Negative	Complies
Storage	Store in cool & dry place. Do not freeze.<br>Keep away from strong light and heat.	
Shelf life	2 years when properly stored.	

*No.*125 *Rehmannia glutinosa* extract

**Product Name**	*Rehmannia glutinosa* extract	
Lot Number	T0306	
Quantity	1500 kg	
Manufacture Date	2022-01-15	
Testing Date	2022-01-19	
**Analysis**	**Specification**	**Results**
Appearance	Yellow-brown powder	Complies
Loss on Drying	≤ 5.0	3.25
Ash	≤ 8.0	3.88
Heavy metals	≤ 10 ppm	Complies
Pb	≤ 2.0 ppm	Complies
As	≤ 2.0 ppm	Complies
Odor	Characteristic	Complies
Particle size	100% through 80 mesh	Complies
**Microbiological**		
Total of bacteria	≤ 3000 cfu/g	Complies
Fungi	≤ 100 cfu/g	Complies
Salmonella	Negative	Complies
E. coli	Negative	Complies
Staph. aureus	Negative	Complies
Storage	Store in cool & dry place. Do not freeze. Keep away from strong light and heat.	
Shelf life	2 years when properly stored.	
