# Supplementary material for: Effects of Helleborus thibetanus extract, a novel plant-derived molluscicide on Oncomelania hupensis, the intermediate host of Schistosoma japonicum
Source: PLoS One. 2026 Sep 11;21(9):e0354674. doi: 10.1371/journal.pone.0354674 (PMC13567751; doi:10.1371/journal.pone.0354674)
Supplement: Supplementary file 2 [file pone.0354674.s001.docx]

**S1 Table. Molluscicidal activity of 125 plant extracts with their corresponding codes**

| No. | Plant extracts | Lot number | Death rate (%) | | | Mean ± SD (%) |
| --- | --- | --- | --- | --- | --- | --- |
|  |  |  | Repeat 1 | Repeat 2 | Repeat 3 |  |
| 1 | *Rhodiola rosea* | T0089 | 10.00 | 10.00 | 20.00 | 13.33 ± 5.77 |
| 2 | *Pausinystalia yohimbe* | T0236 | 10.00 | 0.00 | 10.00 | 6.67 ± 5.77 |
| 3 | *Malpighia emarginata* | T0237 | 20.00 | 10.00 | 10.00 | 13.33 ± 5.77 |
| 4 | *Senna alexandrina* | T0238 | 10.00 | 0.00 | 20.00 | 10.00 ± 10.00 |
| 5 | *Nelumbinis folium* | T0239 | 0.00 | 0.00 | 0.00 | 0.00 ± 0.00 |
| 6 | *Momordica charantia* | T0240 | 0.00 | 0.00 | 10.00 | 3.33 ± 5.77 |
| 7 | *Aloe vera* | T0241 | 10.00 | 10.00 | 0.00 | 6.67 ± 5.77 |
| 8 | *Gentiana scabra* | T0242 | 10.00 | 0.00 | 0.00 | 3.33 ± 5.77 |
| 9 | *Lycium chinense* | T0243 | 20.00 | 0.00 | 0.00 | 6.67 ± 11.55 |
| 10 | *Helianthus tuberosus* | T0244 | 20.00 | 10.00 | 20.00 | 16.67 ± 5.77 |
| 11 | *Folium mori* | T0246 | 20.00 | 0.00 | 20.00 | 13.33 ± 11.55 |
| 12 | *Trifolium pratense* | T0247 | 0.00 | 0.00 | 0.00 | 0.00 ± 0.00 |
| 13 | *Serenoa repens* | T0248 | 0.00 | 10.00 | 0.00 | 3.33 ± 5.77 |
| 14 | *Olea europaea L* | DG0132 | 10.00 | 0.00 | 0.00 | 3.33 ± 5.77 |
| 15 | *Eleutherococcus senticosus* | T0061 | 0.00 | 10.00 | 10.00 | 6.67 ± 5.77 |
| 16 | *Yucca smalliana* | T0253 | 0.00 | 0.00 | 0.00 | 0.00 ± 0.00 |
| 17 | *Valeriana officinalis* | T0256 | 0.00 | 0.00 | 0.00 | 0.00 ± 0.00 |
| 18 | *Semen trigone* | T0249 | 0.00 | 0.00 | 0.00 | 0.00 ± 0.00 |
| 19 | *Garcinia cambogia* | T0258 | 0.00 | 0.00 | 20.00 | 6.67 ± 11.55 |
| 20 | *Coffea arabica* | T0267 | 10.00 | 20.00 | 20.00 | 16.67 ± 5.77 |
| 21 | *Sapindus saponaria* | T0250 | 0.00 | 0.00 | 0.00 | 0.00 ± 0.00 |
| 22 | *Echinacea purpurea* | T0262 | 0.00 | 20.00 | 10.00 | 10.00 ± 10.00 |
| 23 | *Helleborus thibetanus* | DT0221 | 100.00 | 100.00 | 100.00 | 100.00 ± 0.00 |
| 24 | *Garcinia mangostana* | T0266 | 0.00 | 0.00 | 0.00 | 0.00 ± 0.00 |
| 25 | *Eriobotrya japonica* | T0251 | 0.00 | 0.00 | 0.00 | 0.00 ± 0.00 |
| 26 | *Silybum marianum* | T0255 | 10.00 | 10.00 | 20.00 | 13.33 ± 5.77 |
| 27 | *Centella asiatica* | T0227 | 0.00 | 0.00 | 0.00 | 0.00 ± 0.00 |
| 28 | *Salix babylonica* | T0261 | 0.00 | 0.00 | 0.00 | 0.00 ± 0.00 |
| 29 | *Tagetes erecta* | T0259 | 0.00 | 0.00 | 0.00 | 0.00 ± 0.00 |
| 30 | *Arctium lappa* | T0264 | 0.00 | 10.00 | 20.00 | 10.00 ± 10.00 |
| 31 | *Paeonia lactiflora* | T0265 | 0.00 | 0.00 | 20.00 | 6.67 ± 11.55 |
| 32 | *Rosmarinus officinalis* | DM0244 | 10.00 | 0.00 | 20.00 | 10.00 ± 10.00 |
| 33 | *Lampranthus spectabilis* | T0268 | 0.00 | 0.00 | 0.00 | 0.00 ± 0.00 |
| 34 | *Salvia hispanica* | T0269 | 0.00 | 0.00 | 0.00 | 0.00 ± 0.00 |
| 35 | *Annona muricata* | T0270 | 0.00 | 0.00 | 10.00 | 3.33 ± 5.77 |
| 36 | *Taraxacum mongolicum* | T0257 | 0.00 | 0.00 | 0.00 | 0.00 ± 0.00 |
| 37 | *Polygonatum kingianum* | T0260 | 20.00 | 10.00 | 20.00 | 16.67 ± 5.77 |
| 38 | *Cynomorium songaricum* | T0276 | 20.00 | 10.00 | 0.00 | 10.00 ± 10.00 |
| 39 | *Cistanche deserticola* | T0273 | 0.00 | 10.00 | 10.00 | 6.67 ± 5.77 |
| 40 | *Eurycoma longifolia* | T0277 | 0.00 | 0.00 | 0.00 | 0.00 ± 0.00 |
| 41 | *Cuscuta australis* | T0283 | 0.00 | 0.00 | 0.00 | 0.00 ± 0.00 |
| 42 | *Nelumbinis semen* | T0271 | 0.00 | 0.00 | 0.00 | 0.00 ± 0.00 |
| 43 | *Chrysanthemum morifolium* | T0285 | 10.00 | 0.00 | 0.00 | 3.33 ± 5.77 |
| 44 | *Dendrobii caulis* | T0293 | 10.00 | 0.00 | 0.00 | 3.33 ± 5.77 |
| 45 | *Semen hoveniae* | T0307 | 0.00 | 0.00 | 0.00 | 0.00 ± 0.00 |
| 46 | *Phyllanthus emblica* | T0314 | 0.00 | 10.00 | 20.00 | 10.00 ± 10.00 |
| 47 | *Wolfiporia cocos* | T0274 | 10.00 | 0.00 | 30.00 | 13.33 ± 15.28 |
| 48 | *Protea cynaroides* | DP0090 | 0.00 | 0.00 | 0.00 | 0.00 ± 0.00 |
| 49 | *Cassia tora* | T0234 | 0.00 | 0.00 | 0.00 | 0.00 ± 0.00 |
| 50 | *Salvia japonica* | T0294 | 10.00 | 0.00 | 0.00 | 3.33 ± 5.77 |
| 51 | *Fallopia multiflora* | T0338 | 0.00 | 0.00 | 0.00 | 0.00 ± 0.00 |
| 52 | *Opuntia stricta* | T0339 | 0.00 | 10.00 | 0.00 | 3.33 ± 5.77 |
| 53 | *Leonurus artemisia* | T0315 | 0.00 | 0.00 | 20.00 | 6.67 ± 11.55 |
| 54 | *Euphrasia pectinata* | T0340 | 10.00 | 10.00 | 0.00 | 6.67 ± 5.77 |
| 55 | *Plantago asiatica* | T0345 | 0.00 | 0.00 | 0.00 | 0.00 ± 0.00 |
| 56 | *Asparagus cochinchinensis* | T0284 | 0.00 | 0.00 | 0.00 | 0.00 ± 0.00 |
| 57 | *Vitex negundo* | T0327 | 0.00 | 10.00 | 10.00 | 6.67 ± 5.77 |
| 58 | *Morinda officinalis* | T0321 | 0.00 | 10.00 | 0.00 | 3.33 ± 5.77 |
| 59 | *Cyperus rotundus* | T0341 | 0.00 | 0.00 | 0.00 | 0.00 ± 0.00 |
| 60 | *Houttuynia cordata* | T0083 | 0.00 | 10.00 | 0.00 | 3.33 ± 5.77 |
| 61 | *Ajuga ciliata* | T0287 | 0.00 | 20.00 | 10.00 | 10.00 ± 10.00 |
| 62 | *Dictamnus dasycarpus* | T0322 | 0.00 | 10.00 | 0.00 | 3.33 ± 5.77 |
| 63 | *Trigonella foenum-graecum* | T0328 | 0.00 | 10.00 | 0.00 | 3.33 ± 5.77 |
| 64 | *Ficus carica* | T0316 | 0.00 | 0.00 | 10.00 | 3.33 ± 5.77 |
| 65 | *Alpinia oxyphylla* | T0317 | 0.00 | 10.00 | 10.00 | 6.67 ± 5.77 |
| 66 | *Ligusticum sinense* | T0346 | 0.00 | 0.00 | 0.00 | 0.00 ± 0.00 |
| 67 | *Syzygium aromaticum* | T0278 | 0.00 | 10.00 | 0.00 | 3.33 ± 5.77 |
| 68 | *Aucklandiae radix* | T0263 | 0.00 | 0.00 | 0.00 | 0.00 ± 0.00 |
| 69 | *Angelica dahurica* | T0323 | 0.00 | 10.00 | 0.00 | 3.33 ± 5.77 |
| 70 | *Brassica oleracea var. italica* | T0342 | 0.00 | 10.00 | 0.00 | 3.33 ± 5.77 |
| 71 | *Alisma plantago-aquatica* | T0308 | 20.00 | 10.00 | 40.00 | 23.33 ± 15.28 |
| 72 | *Platycodon grandiflorus* | T0288 | 20.00 | 10.00 | 0.00 | 10.00 ± 10.00 |
| 73 | *Cinnamomum cassia* | T0329 | 0.00 | 10.00 | 0.00 | 3.33 ± 5.77 |
| 74 | *Paeonia lactiflora* | T0295 | 0.00 | 0.00 | 0.00 | 0.00 ± 0.00 |
| 75 | *Astragalus membranaceus* | T0330 | 0.00 | 0.00 | 0.00 | 0.00 ± 0.00 |
| 76 | *Scutellariae radix* | T0331 | 0.00 | 0.00 | 0.00 | 0.00 ± 0.00 |
| 77 | *Pueraria lobata* | T0081 | 0.00 | 0.00 | 0.00 | 0.00 ± 0.00 |
| 78 | *Lonicera japonica* | T0289 | 0.00 | 0.00 | 10.00 | 3.33 ± 5.77 |
| 79 | *Zingiber officinale* | T0296 | 10.00 | 10.00 | 20.00 | 13.33 ± 5.77 |
| 80 | *Sophora flavescens* | T0224 | 0.00 | 10.00 | 0.00 | 3.33 ± 5.77 |
| 81 | *Allium sativum* | T0279 | 0.00 | 0.00 | 0.00 | 0.00 ± 0.00 |
| 82 | *Rheum officinale* | T0280 | 20.00 | 0.00 | 0.00 | 6.67 ± 11.55 |
| 83 | *Cnidium monnieri* | T0297 | 10.00 | 10.00 | 0.00 | 6.67 ± 5.77 |
| 84 | *Angelica sinensis* | T0281 | 10.00 | 20.00 | 10.00 | 13.33 ± 5.77 |
| 85 | *Tribulus terrestris* | T0347 | 0.00 | 0.00 | 0.00 | 0.00 ± 0.00 |
| 86 | *Glycyrrhiza uralensis* | T0064 | 0.00 | 10.00 | 20.00 | 10.00 ± 10.00 |
| 87 | *Magnolia officinalis* | T0332 | 0.00 | 10.00 | 0.00 | 3.33 ± 5.77 |
| 88 | *Schisandra chinensis* | T0326 | 0.00 | 10.00 | 0.00 | 3.33 ± 5.77 |
| 89 | *Lycium ruthenicum* | T0333 | 0.00 | 0.00 | 0.00 | 0.00 ± 0.00 |
| 90 | *Gynostemma pentaphyllum* | T0290 | 0.00 | 0.00 | 0.00 | 0.00 ± 0.00 |
| 91 | *Hypericum perforatum* | T0286 | 0.00 | 10.00 | 10.00 | 6.67 ± 5.77 |
| 92 | *Citrus aurantium* | T0309 | 0.00 | 0.00 | 0.00 | 0.00 ± 0.00 |
| 93 | *Equisetum arvense* | T0291 | 0.00 | 10.00 | 0.00 | 3.33 ± 5.77 |
| 94 | *Gardenia jasminoides* | T0311 | 10.00 | 10.00 | 0.00 | 6.67 ± 5.77 |
| 95 | *Punica granatum* | T0298 | 10.00 | 10.00 | 10.00 | 10.00 ± 0.00 |
| 96 | *Trifolium pratense* | T0334 | 0.00 | 0.00 | 20.00 | 6.67 ± 11.55 |
| 97 | *Camellia sinensis* | T0351 | 10.00 | 0.00 | 0.00 | 3.33 ± 5.77 |
| 98 | *Phaseolus vulgaris* | DB0358 | 0.00 | 10.00 | 0.00 | 3.33 ± 5.77 |
| 99 | *Brassica oleracea* | T0318 | 0.00 | 0.00 | 0.00 | 0.00 ± 0.00 |
| 100 | *Prunella vulgaris* | T0343 | 0.00 | 0.00 | 20.00 | 6.67 ± 11.55 |
| 101 | *Ziziphi spinosae* | T0299 | 0.00 | 10.00 | 0.00 | 3.33 ± 5.77 |
| 102 | *Cannabis fructus* | T0335 | 0.00 | 0.00 | 0.00 | 0.00 ± 0.00 |
| 103 | *Fructus mori* | T0300 | 0.00 | 10.00 | 0.00 | 3.33 ± 5.77 |
| 104 | *Ramulus mori* | T0301 | 0.00 | 0.00 | 0.00 | 0.00 ± 0.00 |
| 105 | *Rhamnus utilis* | T0302 | 0.00 | 0.00 | 0.00 | 0.00 ± 0.00 |
| 106 | *Passiflora caerulea* | T0344 | 0.00 | 0.00 | 0.00 | 0.00 ± 0.00 |
| 107 | *Ophiopogon japonicus* | T0272 | 0.00 | 0.00 | 0.00 | 0.00 ± 0.00 |
| 108 | *Coix lacryma-jobi* L. var*. ma-yuen* | T0319 | 0.00 | 0.00 | 20.00 | 6.67 ± 11.55 |
| 109 | *Rehmannia glutinosa* | T0303 | 10.00 | 10.00 | 20.00 | 13.33 ± 5.77 |
| 110 | *Ziziphus jujuba* | T0282 | 0.00 | 0.00 | 0.00 | 0.00 ± 0.00 |
| 111 | *Phellodendron chinense* | T0336 | 0.00 | 0.00 | 0.00 | 0.00 ± 0.00 |
| 112 | *Dichroae radix* | T0348 | 0.00 | 10.00 | 20.00 | 10.00 ± 10.00 |
| 113 | *Atractylodes macrocephala* | T0352 | 10.00 | 10.00 | 30.00 | 16.67 ± 11.55 |
| 114 | *Bletilla striata* | T0353 | 0.00 | 0.00 | 0.00 | 0.00 ± 0.00 |
| 115 | *Agastache rugosa* | T0337 | 0.00 | 10.00 | 0.00 | 3.33 ± 5.77 |
| 116 | *Xanthium strumarium* | T0349 | 0.00 | 0.00 | 0.00 | 0.00 ± 0.00 |
| 117 | *Citrus reticulata* | T0350 | 0.00 | 0.00 | 20.00 | 6.67 ± 11.55 |
| 118 | *Coptis chinensis* | T0354 | 0.00 | 0.00 | 30.00 | 10.00 ± 17.32 |
| 119 | *Apium graveolens* | T0355 | 0.00 | 10.00 | 0.00 | 3.33 ± 5.77 |
| 120 | *Callerya reticulata* | T0292 | 0.00 | 0.00 | 0.00 | 0.00 ± 0.00 |
| 121 | *Panax notoginseng* | T0304 | 0.00 | 0.00 | 0.00 | 0.00 ± 0.00 |
| 122 | *Cornus officinalis* | T0305 | 0.00 | 10.00 | 0.00 | 3.33 ± 5.77 |
| 123 | *Saposhnikovia divaricata* | T0275 | 0.00 | 10.00 | 10.00 | 6.67 ± 5.77 |
| 124 | *Perilla frutescens* | T0313 | 0.00 | 0.00 | 0.00 | 0.00 ± 0.00 |
| 125 | *Rehmannia glutinosa* | T0306 | 0.00 | 20.00 | 0.00 | 6.67 ± 11.55 |
